# Cardiovascular health impacts of wildfire smoke exposure

**DOI:** 10.1186/s12989-020-00394-8

**Published:** 2021-01-07

**Authors:** Hao Chen, James M. Samet, Philip A. Bromberg, Haiyan Tong

**Affiliations:** 1 Oak Ridge Institute for Science and Education, Oak Ridge, TN 37830 USA; 2 Public Health and Integrated Toxicology Division, Center for Public Health and Environmental Assessment, U.S. Environmental Protection Agency, Chapel Hill, NC 27514 USA; 3 Center for Environmental Medicine, Asthma and Lung Biology, University of North Carolina at Chapel Hill, Chapel Hill, NC 27514 USA

**Keywords:** Wildfire smoke, Wood smoke, Air pollution, Cardiovascular health

## Abstract

In recent years, wildland fires have occurred more frequently and with increased intensity in many fire-prone areas. In addition to the direct life and economic losses attributable to wildfires, the emitted smoke is a major contributor to ambient air pollution, leading to significant public health impacts. Wildfire smoke is a complex mixture of particulate matter (PM), gases such as carbon monoxide, nitrogen oxide, and volatile and semi-volatile organic compounds. PM from wildfire smoke has a high content of elemental carbon and organic carbon, with lesser amounts of metal compounds. Epidemiological studies have consistently found an association between exposure to wildfire smoke (typically monitored as the PM concentration) and increased respiratory morbidity and mortality. However, previous reviews of the health effects of wildfire smoke exposure have not established a conclusive link between wildfire smoke exposure and adverse cardiovascular effects. In this review, we systematically evaluate published epidemiological observations, controlled clinical exposure studies, and toxicological studies focusing on evidence of wildfire smoke exposure and cardiovascular effects, and identify knowledge gaps. Improving exposure assessment and identifying sensitive cardiovascular endpoints will serve to better understand the association between exposure to wildfire smoke and cardiovascular effects and the mechanisms involved. Similarly, filling the knowledge gaps identified in this review will better define adverse cardiovascular health effects of exposure to wildfire smoke, thus informing risk assessments and potentially leading to the development of targeted interventional strategies to mitigate the health impacts of wildfire smoke.

**Supplementary Information:**

The online version contains supplementary material available at 10.1186/s12989-020-00394-8.

## Introduction

Wildland fires have been an important factor in shaping the landscape and controlling the biomes on Earth for millions of years [115]. Over recent decades, rapid growth of the wildland-urban interface and anthropogenic climate change have contributed to increased wildfire frequency and unprecedented intensity in many fire-prone regions [1, 22, 117, 163, 169]. In the western United States, summer wildfire frequency increased approximately eightfold between 1972 and 2018; resulting in a fivefold rise in the annual area burned [163]. Southeastern Australia is a densely populated region where large-scale wildfires have led to massive life and property loss [169]. Multiple forms of vegetation in wildlands, including trees, bushes, grass, and their partially decayed form “peat”, fuel these fires, and smoke emitted from the combustion of these vegetations are referred to as wildfire smoke, with alternative names including “landscape fire smoke”, “wildland fire smoke”, “peat fire smoke”, “forest fire smoke”, and “biomass smoke” [30, 75, 156]. Wildfire smoke contains many air pollutants known to be detrimental to human health, such as particulate matter (PM) and volatile organic compounds (VOCs) [4, 30].

Wildfires impact public health since wildfire smoke presents a source of air pollution in neighboring and even more distant populated areas [30, 85]. Epidemiological studies estimate that the global mortality burden attributable to landscape fire smoke is approximately 339,000 deaths annually [75]. Between 2008 to 2012, about 10.3 million individuals in the United States were estimated to have experienced unhealthy air quality levels (average daily fire-PM_2.5_ > 35 μg/m^3^) associated with exposure to wildfire for more than 10 days [120]. Evidence also indicates an association between the long-distance transport of PM_2.5_ resulting from wildfire smoke and the adverse health effects in susceptible populations thousands of miles away [84, 85].

As a leading cause of death worldwide [69], cardiovascular disease (CVD), especially ischemic heart disease (IHD) and stroke, has been shown to associate with increased levels of air pollutants such as PM [51]. Wildfire events can cause an abrupt increase in ambient PM_2.5_ levels to more than 2000 μg/m^3^ [3, 8]. Exposure to such short-term elevations in PM levels are capable of evoking cardiac arrhythmias, worsening heart failure, and triggering atherosclerotic/ischemic cardiovascular complications, particularly in certain high-risk subpopulations [29]. Biomass smoke has also been found to be comparable to tobacco smoke as a cardiovascular comorbidity factor among patients with COPD, suggesting the need to treat diseases associated with biomass combustion similarly to those associated with tobacco use [61].

Understanding whether wildfire smoke exposure increases cardiovascular risk is of utmost importance to provide scientific evidence to better inform policymaking to protect public health, especially in the most vulnerable segments of the population. Wildfire smoke exposure has been linked to increased hospitalization and emergency department (ED) visits for respiratory symptoms, exacerbation of asthma, and chronic obstructive pulmonary disease (COPD), but the association with elevated cardiovascular risks, primarily based on epidemiological studies, is less conclusive [4, 30, 123, 124]. In recent years, a growing number of observational and experimental studies have investigated cardiovascular effects of wildfire smoke and their associated mechanisms. The observational studies include studies focusing on wildfire smoke and also on household biomass smoke that can be important sources of evidence of an association with cardiovascular risk [47]. The experimental studies using wood smoke, are more likely to capture acute pathophysiological changes associated with wood smoke exposure, compared to the observational studies that are based on healthcare data such as hospitalizations and ED visits for acute cardiovascular symptoms [138]. In this review, we will systematically examine the evidence from these studies on the association between cardiovascular health impacts and wildfire smoke (including biomass smoke and wood smoke) exposure and their possible underlying mechanisms. This review will also identify knowledge gaps and point to possible directions for future research.

## Literature search

Wildfire in this review is conceptualized within the definitions of landscape fires encompassing wild and prescribed forest fires, tropical deforestation fires, peat fires, agricultural burning, and grass fires. Wildfires release approximately 2 × 10^12^ kg of carbon into the atmosphere annually [75, 156]. The main aim of this review is to examine evidence from the published peer-reviewed literature on the association between cardiovascular effects and exposure to wildfire smoke. Combustion of household biomass fuels for cooking and heating including wood, grass, and straw, which are also commonly found in wildfire scenarios, contributes to both indoor and ambient air pollution. In addition, a number of controlled human wood smoke exposure studies that are sufficiently powered to infer a causal relationship will be discussed in concert with both in vivo animal and in vitro toxicological experiments. Although the composition of household biomass smoke and experimental wood smoke is different from that of wildfire smoke, studies of biomass and experimental wood smoke provide important information that may be used to infer an association between cardiovascular effects and wildfire smoke and the associated mechanisms. Therefore, a number of household biomass smoke studies as well as clinical and experimental wood smoke studies will also be included to complement the wildfire studies in this review.

**Fig. 1:**
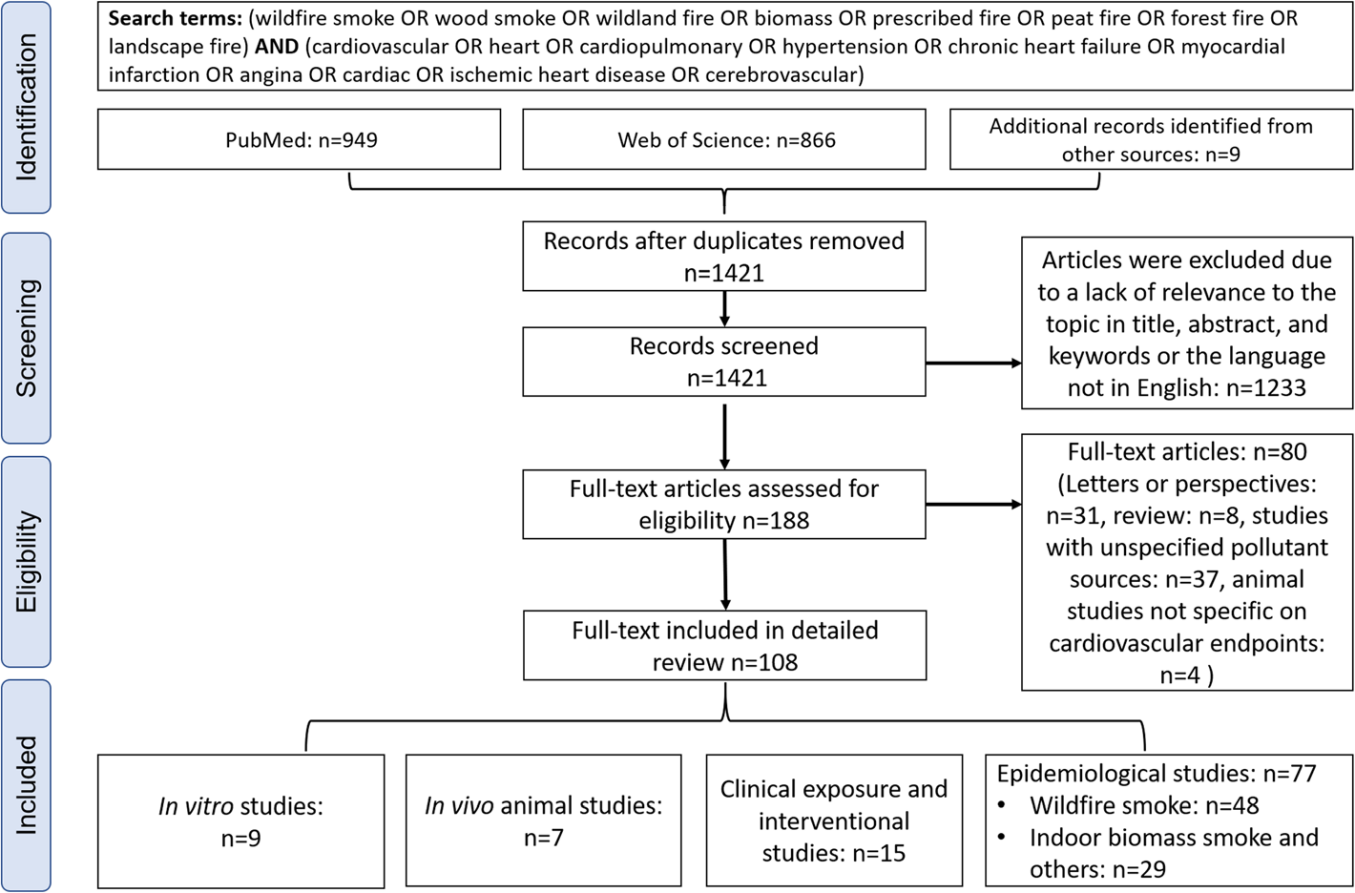
Literature review flow chart

We searched peer-reviewed publications on the topic of wildfire smoke and cardiovascular health covering the period between 1980 and 2020. Terms including “forest fire smoke”, “wildfire smoke”, “biomass smoke”, “wildland fire smoke”, “peat fire smoke”, “wood smoke”, and “prescribed fire” have been employed interchangeably in the literature. Searches were performed using two engines, “PubMed” and “Web of Science”. As shown in Fig. 1, searching “PubMed” and “Web of Science” yielded 949 and 866 results as of April 7th, 2020, respectively. After duplicate exclusion and title screening, 182 records in English language were assessed for their eligibility for further review. After excluding 31 letters or perspectives, 8 review articles, and 37 studies on coal or animal dung as combustion fuel or that did not specify the types of fuel, 108 publications were included for detailed review. These included 77 epidemiological studies which were further grouped into 48 wildfire smoke studies (Table 2 and Table S1) and 29 indoor and ambient biomass smoke studies (Table S2). Of the remaining 31 articles, 15 were controlled human exposure or interventional studies (Table S3), 7 were in vivo animal studies (Table S4), and 9 were in vitro studies (Table S5).

## Composition of wildfire smoke

Wildfire smoke is complex physically and chemically, and its composition is determined by fuel type and combustion conditions [30, 83]. Wildfire smoke generally consists of coarse and fine PM, VOCs (e.g., aldehydes, n-alkanes), polycyclic aromatic hydrocarbons (PAHs), gases (e.g., CO, SO_2_, NO, NO_2_), and metals [155]. PM composition is a summation of the emitted mixture of compounds that is usually present as soot or oily substances high in elemental and organic carbon, and metallic compounds [155]. VOCs and gases are normally dispersed into the environment and they are able to react further photochemically to generate secondary organic aerosol (SOA), for example in situ ozone oxidation of alkanes in the ambient air, which can also be detrimental to human health [60, 113]. Clinical and toxicological studies offer a detailed opportunity to examine the chemical compositions of wood smoke, a major contributor of wildfire smoke, in a controlled experimental setting. The chemical composition of wood smoke in a laboratory setting is different from that of wildfire smoke in a natural environment where vegetation characteristics, combustion conditions, weather conditions, and the geographical area burnt are factors that add complexity relative to emissions from the combustion or pyrolysis of one or two types of wood in a controlled chamber environment. As summarized from these studies in Table 1, wood smoke, independently of fuel type and combustion conditions, is characterized by the presence of PM, gases, n-alkanes, PAHs, methoxyphenol compounds, levoglucosan, and metals species.

**Table 1:** Chemicals detected in wood smoke in laboratory studies

Pollutant category	Chemicals found in the wood smoke	Note
Particulate matter	PM_1_, PM_2.5_, PM_10_	The mass of PM ranges from tens to thousands in μg/m^3 a^
Gases	CO, NO, NO_2_, SO_2_	CO: 2.7–1385 ppm ^a^
n-Alkane compounds	Pentadecane, Hexadecane, Octadecane, Nonadecane, Docosane, Tricosane, Tetracosane, Pentacosane, Heptacosane	% n-Alkane in PM mass: 0–0.9% ^a^
Polyaromatic compounds	Phenanthrene, Anthracene, Methylphenanthrene, Methylanthracene, Fluoranthene, Pyrene, Benz(a)fluorene, Benz(b)fluorene, Methylchrysene, Naphthalene, Retene, Chrysene,	% PAH compounds in PM mass: 0–0.5% ^a^
Methoxyphenol compounds	Guaiacol, Methoxymethyl phenol, Ethylguaiacol, Eugenol, Propylguaiacol, Vanillin, Isoeugenol, 3,5-Dimethoxyphenol, Syringaldehyde, Acetosyringone, Coniferyl aldehyde	% Methoxyphenol in PM mass: 0.1–6.5% ^a^
Levoglucosan	Levoglucosan	% Levoglucosan in PM mass: 0.9–12.6% ^a^
Inorganic constituents	Ca, Cd, Cr, Cu, K, Mg, Na, Pb, Si, Zn, Al, Ba, Fe, Mn, NH_4_^+^, PO_4_^3−^, Cl^−^, NO_3_^−^, SO_4_^2−^,	–

Note: Representative chemicals and their values were adapted and summarized from published studies ([17, 57, 58, 83, 90, 100, 106]). Note: ^a^ indicated the detected range dependent on variations in wood types (e.g. red oak, birch, eucalyptus, peat, etc) and combustion conditions (e.g., incomplete versus complete)

Most epidemiological studies in this review did not provide detailed chemical profiles of the wildfire smoke studied. Instead, they employed large-scale air pollution data derived from local air quality monitoring stations or satellite imagery obtained during wildfire seasons that feature elevated levels of PM_2.5_ and/or PM_10_, with a few studies also relying on the concentrations of other index air pollutants such as SO_2_, oxides of nitrogen (NO_x_), CO, and ozone [2, 28, 37, 43, 45, 92, 126]. In all cases, wildfire events dramatically elevated ambient air pollutant levels. For example, the peak levels of PM_2.5_ and PM_10_ during wildfire days were approximately 10 times higher than those in non-fire days in Sydney, Australia [94]. Efforts have also been made specifically to address the role of organic compounds in epidemiology by using environmental markers of PAHs such as delta-C [18, 19, 38, 45, 127]. Delta-C is based on a calculation of the difference between light absorption of ultraviolet black carbon (BC), measured at 370 nm and BC 880 nm with a two-wavelength aethalometer [38], and was used for source apportionment for wood smoke [159]. Studies have employed urinary 1-hydroxypyrene (1-OHP), a metabolic biomarker of PAHs [14, 131, 132] as an index of exposure, and found that the urinary 1-OHP levels measured in a group of Mexican women who were exposed to indoor biomass smoke can be 11 times above the mean reported for the general population [132]. In addition, measurement of levoglucosan, a sugar anhydride byproduct of pyrolysis of cellulose was utilized as a relative indicator of exposure to products of pyrolysis from burning biomass [55].

## Cardiovascular effects of wildfire smoke

### Epidemiological studies

Wildfire events are normally transient in nature and, therefore, pose a challenge to the implementation of prospective cohort or case-control epidemiological study designs that require long-term exposure observations. A significant challenge is the lack of clear definition of the timeline that corresponds to the PM measurements, for instance, whether it represent 3-day averages, 24 h averages or one-hour daily peak averages. Instead, cross-sectional, case crossover time-series, population-based cohort, or ecological study designs are commonly employed to assess the possible association between wildfire smoke exposure and cardiovascular risk in epidemiological studies as summarized in Table 2 and Table S1. Most of these studies have found that certain populations are at a higher risk of acute adverse cardiovascular effects due to wildfire smoke exposure.

**Table 2:** A summary of epidemiological studies of the association between wildfire smoke exposure and cardiovascular effects

Study	Exposure measurement	Endpoints	Association
**Cardiovascular morbidity**			
Mott et al. [105]	Smoky vs. non-smoky periods	Overall cardiorespiratory	↑
		IHD	→
		Circulatory disease	→
Moore et al. [103]	Temporal comparison	All CVD	→
Johnston et al. [73]	Monitored PM_10_	All CVD	→
		IHD	↑
Hanigan, Johnston, and Morgan [65]	Modeled PM_10_	All circulatory	→
		IHD	→
Delfino et al. [41]	Estimated PM_2.5_ and PM_10_	All CVD	→
		IHD	→
		HF	→
		Other	→
Henderson et al. [68]	Modeled PM_10_	All CVD	→
Lee et al. [86]	Monitored PM_10_	Circulatory	↑
		CAD	↑
Morgan et al. [104]	Monitored PM_10_	All CVD	↑
		Cardiac	↑
		IHD	→
		Stroke	→
Schranz, Castillo, and Vilke [137]	Temporal comparison	CAD	→
		CHD	→
Henderson et al. [67]	Modeled PM_10_	All CVD	→
Rappold et al. [121]	Temporal and spatial comparison	All CVD	→
		MI	→
		HF	↑
Crabbe [37]	Monitored PM_10_, coarse PM, fine PM, black carbon	CVD	↑
Rappold et al. [118]	Modeled PM_2.5_	Congestive HF	↑
Hejl et al. [66]	Temporal comparison	Blood cardiovascular markers	↑
Martin et al. [94]	Smoky vs. non-smoky periods	Arrythmia	→
		Cardiac failure	→
		Cerebrovascular disease	→
		IHD	→
Gaughan et al. [55]	Urinary levoglucosan level	Mean augmentation index %	↑
Rappold et al. [119]	Modeled PM_2.5_	Congestive HF	↑
Johnston et al. [76]	Smoky vs. non-smoky periods	All CVD	→
		IHD	↑
		Arrythmias	↑
		Cerebrovascular disease	→
		Cardiac failure	↑
Le et al. [85]	Temporal comparison, monitored PM_2.5_	All CVD	↑
		IHD	↑
		HF	↑
		Cerebrovascular disease	→
		Heart rate disturbances	↑
Dennekamp et al. [43]	Monitored PM_2.5_, PM_10_, CO, O_3_, SO_2_, NO_2_	Cardiac arrest	↑
Haikerwal et al. [64]	Modeled PM_2.5_	Cardiac arrest	↑
		IHD	↑
		Acute MI	→
		Angina	→
Resnick et al. [126]	Temporal comparison	All CVD	↑
		IHD	→
		Hypertension	→
		Cerebrovascular disease	↑
		Disease of veins, lymphatics, and circulatory system	↑
Alman et al. [8]	Monitored PM_2.5_	CVD	→
		Acute MI	→
		IHD	→
		Dysrhythmia	→
Ostro et al. [111]	Modeled source-apportionment PM_2.5_	All CVD	→
		IHD	→
		MI	→
		Dysrhythmia	↑
		HF	→
Reid, Jerrett, et al. [123, 124]	Temporal comparison and modeled PM_2.5_	All CVD	→
		IHD	→
		CHD	→
		Congestive HF	→
Tinling et al. [149]	Modeled PM_2.5_	All-cause cardiac conditions	↑
		Cardiac dysrhythmia	↑
		HF	→
		Hypertension	↑
Yao, Eyamie, and Henderson 2016) [165]	Smoky vs. non-smoky periods	CVD	↑
Gan et al. [53]	Modeled PM_2.5_	CVD	↑
		Arrhythmia	→
		Cerebrovascular disease	↑
		HF	→
		IHD	→
		MI	→
Garcia-Olivé et al. [54]	Burnt area % (spatial comparison)	Acute MI	→
		HF	→
		CVD	→
Liu et al. [88]	Temporal comparison and modeled PM_2.5_		
Parthum, Pindilli, and Hogan [114]	Modeled PM_2.5_	CHF	↑
		Cardiopulmonary disease	↑
Salimi et al. [133]	Monitored PM_2.5_	Cardiac arrest	↑
		Stroke	↑
		Other heart problems	↑
Weichenthal et al. [160]	Monitored PM_2.5_	MI	↑
Wettstein et al. [161]	Smoke density	All CVD	↑
		Hypertension	↑
		IHD	→
		MI	↑
		Dysrhythmia	↑
		HF	↑
		Ischemic stroke	↑
Abdo et al. [2]	Modeled PM_2.5_	Gestational hypertension	↑
Deflorio-Barker et al. [40]	Smoke vs non-smoke days	Cardiopulmonary	→
Stowell et al. [147]	Modeled PM_2.5_	Acute MI	→
		Congestive HF	→
		Dysrhythmia	→
		IHD	→
		Cerebrovascular disease	→
		CVD	→
Jones et al. [77]	Modeled PM_2.5_	Cardiac arrest	↑
**Cardiovascular mortality**			
Vedal and Dutton [157]	Monitored PM_2.5_ and PM_10_	Cardiorespiratory	→
Analitis, Georgiadis, and Katsouyanni [10]	Areas burnt (spatial comparison)	CVD	↑
Marlier et al. [92]	Modeled PM_2.5_ and O_3_	CVD	↑
Nunes, Ignotti, and Hacon [109]	Modeled PM_2.5_	CVD	↑
		Acute MI	↑
		Cerebrovascular disease	→
Shaposhnikov et al. [143]	Monitored PM_10_	IHD	↑
		Cerebrovascular disease	↑
Linares et al. [87]	Temporal comparison, monitored PM_2.5_ and PM_10_	Circulatory disease	→
Faustini et al. [48]	Smoky vs. non-smoky days	CVD	↑
Kollanus et al. [84]	Monitored PM_2.5_	CVD	↑
Navarro et al. [107]	Modeled PM_2.5_	CVD	↑
Uda, Hein, and Atmoko [153]	Modeled PM_2.5_	CVD	↑

*Abbreviations*: *CAD* Coronary artery disease, *CHD* Congestive heart disease, *CVD* Cardiovascular disease, *HF* Heart failure, *IHD* Ischemic heart disease, *MI* Myocardial infarction. “↑” indicates a positive association; “→” indicates a null association

#### Cardiovascular morbidity

Of the 48 wildfire epidemiological studies, 38 are on cardiovascular morbidity (Table 2 and S1), and most outcomes focus on ED visits and hospitalizations for cardiovascular symptoms and illness, for which extensive data are available from hospitals and governmental health agencies. Twenty-five of the 38 studies have reported a positive association between wildfire smoke exposure and increased healthcare needs for CVD.

Wildfire smoke exposures were categorized based on exposure time or location. Employing a time-series study design, researchers in Australia stratified continuous air pollution data into different exposure doses in order to compare the healthcare needs between non-smoky days and smoky days when either PM_2.5_ or PM_10_ concentrations exceeded 99% of the days during the entire time series. They found that increased ED visits for CVD (e.g., IHD, arrhythmias) were associated with elevated levels of PM_10_ and PM_2.5_ on smoky days [37, 76]. Lee et al. [86] demonstrated that during the 1999 forest fire near the Hoopa Valley Indian reservation in California, daily PM_10_ levels were significant predictors of the number of patients seeking care for circulatory and respiratory illnesses among residents of Hoopa and nearby communities. A recent study found that out-of-hospital cardiac arrest risk was significantly elevated in association with heavy smoke during wildfire events in California between 2015 to 2017 [77]. Rappold and colleagues employed a population-based cohort study design and discovered that, compared with wildfire-free counties, there was an increased number of ED visits for cardiovascular illness in counties impacted by exposure to smoke from a peat bog wildfire in North Carolina in 2008, with the association being more prominent for the elderly population [121]. Parthum et al. [114] investigated effects of another peatland fire in Virginia in 2008 and found that the fire event significantly contributed to 32 and 33 more ED visits for congestive heart failure (CHF) and cardiopulmonary symptoms respectively, compared with those on non-fire days.

Continuous air pollution data, such as ambient concentration of PM_2.5_ levels, were also commonly directly employed in the analyses. Similar to the North Carolina peat wildfire study [121], a 10 μg/m^3^ increase in 24-h PM_2.5_ during peat fires was correlated with increased risk of hypertension in adults, and of all-cause cardiac outcomes in youth [149]. Haikerwal et al., reported that an interquartile range increase of 9.04 μg/m^3^ in PM_2.5_ over a 2-day moving average during the 2006–2007 wildfire season in Victoria, Australia, was significantly associated with a 6.98% increase in risk of out-of-hospital cardiac arrests, with the strongest associations observed among men and older adults, and was also associated with an increased risk for IHD-related ED visits (2.07%) and hospitalizations (1.86%), particularly among women and the elderly [64]. In addition to PM_2.5_, the same group found that the ambient levels of PM_10_ and CO were also correlated with a 48-h lag to an elevated incidence of increased out-of-hospital cardiac arrests among men during the wildfire events, although PM_2.5_ was still the main contributor of these effects [43]. A larger scale study in the Greater Sydney Metropolitan area from 2004 to 2015 showed similar results, specifically that a 10 μg/m^3^ increase in PM_2.5_ resulting from the wildfire smoke was positively associated with the number of same-day emergency ambulance dispatches responding to calls for cardiac arrest, stroke, and chest pain [133].

In contrast, 13 of the 38 studies reported null associations between cardiovascular healthcare needs and wildfire smoke exposure. During a 2003 forest fire event in British Columbia Canada, despite significant increases in physician visits for respiratory complaints, the physician visits for CVD were not significantly increased by forest fire smoke containing a peak of 200 μg/m^3^ PM_10_ and 250 μg/m^3^ PM_2.5_ [103]. Similar to the British Columbia study, Henderson and colleagues employed a population-based cohort methodology and multiple exposure assessment metrics, including tapered element oscillating microbalance measurements, a CALPUFF dispersion model, and a SMOKE exposure metric. Despite more novel methods, they reached the same conclusion that no significant association were observed between PM_10_ exposure and cardiovascular hospital admissions [67, 68]. Null cardiovascular results were also reported in the USA [8, 40, 41, 88, 123, 124, 137, 147] and in Australia [94, 104], in spite of the presence of strong links to the incidence of respiratory diseases [8, 40, 41, 104, 137, 147].

#### Cardiovascular mortality

Among the 10 epidemiological studies investigating the association between exposure to wildfire smoke and cardiovascular mortality, eight found a positive association. Using satellite-derived estimates and atmospheric modeling for exposure categorization, wildfire contributed to 200 days per year in which the World Health Organization’s 24-h PM_2.5_ interim air quality target was exceeded in Southeast Asia between 1997 and 2006, leading to an estimated 2% annual increase in adult cardiovascular mortality [92]. Similarly, smoky days with PM_10_ higher than 8 μg/m^3^ based on satellite data, were significantly related to increased cardiovascular mortality, during forest fire episodes in several Mediterranean cities in 2003–2010 [48]. A recent study from Indonesia found that peatland fire smoke contributed to an annual average of 26 μg/m^3^ PM_2.5_ per year, and was associated with excess mortality due to respiratory or cardiovascular causes [153]. In addition to PM, other meteorological conditions, such as high temperature, were determined to be co-contributors to the association between increased cardiovascular mortality and wildfire events [143].

As with the morbidity studies discussed above, null associations have also been reported in a couple of cardiovascular mortality studies. One study showed that the daily cardiorespiratory mortality was not associated with same-day wildfire smoke containing peak PM_10_ and PM_2.5_ levels of 372 and 200 μg/m^3^, respectively [157]. Another study also reported no significant associations between increased PM_10_ levels during biomass fires and cardiovascular mortality, even though there was a significant finding for respiratory mortality among people who were ≥ 75 years old [87]. Some authors argue that the mortality impact of short-term increases in PM due to wildfire would not be expected to be limited to 1 day but show a lag several days later [157], and this could explain the lack of a relationship between wildfire smoke exposures and cardiovascular mortality.

#### Occupational exposure to wildfire smoke

Firefighters are a special group of workers who are likely to be exposed to high levels of air pollutants during wildfire events [110, 125]. Hejl et al. [66] showed that firefighters exposed to up to 1306 μg/m^3^ PM_2.5_ during firefighting had increased serum markers of inflammation (IL-8) and altered vascular function (ICAM1, VCAM1). Another study showed that elevated mean augmentation index, an indicator of arterial stiffness, was linked to urinary levoglucosan levels among wildfire firefighters [55]. These pathophysiological changes may lead to higher risks of adverse cardiovascular outcomes among firefighters due to exposure to wildfire smoke. Navarro found that firefighters who worked 49 days per year were exposed to a mean concentration of PM_4_ at 510 μg/m^3^ during wildland firefighting activities. They estimated that the increased PM exposure was associated with increased risk of lung cancer as well as CVD-related mortality [107].

#### Indoor and ambient biomass smoke

While the focus of this review is on wildfire smoke, there are 29 studies (Table S2) examining the health impacts of indoor or ambient exposure to biomass smoke from household fuel combustion for cooking or heating using wood, grass, or straw. Using a cohort study design, researchers identified differences among cardiovascular outcomes between cohorts exposed to biomass smoke and control groups. Compared with those using cleaner fuels, biomass use in Peruvian households was associated with significantly higher levels of indoor PM_2.5_ concentrations, which were, in turn, associated with increased carotid intima media thickness, elevated prevalence of carotid plaques, and higher systolic blood pressure (SBP) [112]. Similarly, the use of biomass fuel for cooking was associated with elevations in SBP, diastolic blood pressure (DBP), and mean arterial pressure, with a stronger association observed among older populations in China [21, 42]. In addition to PM_2.5_, one study in Western Kenya showed that smoke from the incomplete combustion of locally acquired wood contained high levels of household CO, which was a driver of an association with heart abnormalities detected by echocardiography [5]. A cross-sectional study in Turkey reported that long-term exposure to indoor biomass smoke was associated with systolic and diastolic biventricular dysfunction, as well as increased pulmonary artery systolic pressure [80]. However, some studies have found no significant association between increased cardiovascular risk and indoor exposure to biomass smoke [33, 101, 102, 142]. Indoor biomass combustion contributes to ambient air pollution, which has also been linked to increased incidence of cardiovascular outcomes. A 10 μg/m^3^ increase in ambient PM_2.5_ levels due to wood combustion for winter heating in Tasmania, Australia was significantly associated with an increased rate of hospitalization for heart failure [72]. Domestic wood combustion for heating in Central Launceston, Australia was associated with increased levels of ambient PM_10_ and increased cardiovascular mortality; the effects were mitigated by intervention to reduce wood smoke production [74]. It is important to note that smoke emitted from household combustion of biomass is not equal to wildfire smoke; therefore, caution is advised when interpreting results from these biomass smoke studies.

#### Vulnerable populations

It is known that certain subpopulations are more susceptible to health impacts of exposure to air pollution, including children, the elderly, the obese, pregnant women, people with low socio-economic status (SES), and those with underlying lung and heart diseases [79, 96]. These subpopulations are also more susceptible to wildfire smoke exposure, especially the elderly, pregnant women, and those with lower SES.

The elderly are more likely to seek healthcare (i.e. ED visits, hospitalizations) due to cardiovascular issues, such as IHD, CHF, cardiac arrest and acute myocardial infarction (MI), during wildfire season [64, 76, 77, 105, 118]. Many more studies are in agreement with this finding, with small variations of the age at which these effects are evident. Exposure to high PM_2.5_ levels were strongly associated with increased rates of acute MI among people > 75 years old [109], and with increases in ED visits for cardiovascular dysfunction (e.g. IHD, dysrhythmia, heart failure, stroke and MI) among people > 65 years old [126, 161]. In addition, meteorological factors such as low ambient temperature positively modified the association between increases in 3-day average PM_2.5_ levels and increased risk of MI among the elderly (≥ 65 years old) during biomass fire periods [160]. Interestingly, a few studies revealed the possibility that wildfire emissions can impact the health of more remote elderly populations after long-distance transport of the particles. The 2002 Quebec forest fires in Canada significantly increased average PM_2.5_ levels during hazy days to 53 μg/m^3^ from 21.5 μg/m^3^ on clear days in the United States. Compared with the clear days, the rate of hospitalizations for both respiratory and cardiovascular illness were increased during the hazy days among the elderly [85]. Similar evidence was found in a Finnish study which reported that a 10 μg/m^3^ increase in PM_2.5_, attributed to a long-range transport from vegetation fires, was associated with all-cardiovascular mortality especially among population > 65 years old [84].

Pregnant women are also susceptible to wildfire smoke exposure. Abdo and colleagues examined the impact of wildfire smoke on hypertension during pregnancy and reported that a 1 μg/m^3^ increase in PM_2.5_ exposure to wildfire smoke over the full gestation or during the 1st and 2nd trimester was positively associated with gestational hypertension, in addition to adverse effects on premature birth and decreased birth weight in newborns [2]. Using delta-C as an environmental marker for wood combustion, Assibey-Mensah and colleagues first discovered that each 0.52 μg/m^3^ increase in delta-C concentration during the 7th and 8th gestational month was associated with an elevated risk of hypertensive disorders of pregnancy [18]. They also report that exposure to wood smoke-sourced fine PM was positively associated with an increased risk of early onset of preeclampsia during pregnancy [19].

Health disparity was another risk factor that may render certain subpopulations more susceptible to wildfire smoke-associated cardiovascular effects. A study showed that indigenous people in Darwin, Australia were more likely to be admitted to the hospital for IHD and respiratory illnesses during vegetation fire season when there were significantly increased levels of PM_10_ [65, 73]. Jones and colleagues indicated that low SES may increase the risk of out-of-hospital cardiac arrest due to wildfire smoke exposure during wildfire events in California [77]. Rappold and colleagues compared the ED visits due to wildfire smoke between top- and bottom-ranked counties in North Carolina stratified by socioeconomic factor and found that increased ED visits for asthma and CHF were significantly higher in the low SES counties, 85 and 124% respectively, per 100 μg/m^3^ increase in PM_2.5_ [118].

Cigarette smoking may increase the susceptibility to adverse health impacts of wildfire smoke among smokers. Cigarette smoking is an established risk factor for cardiovascular disease [9] and there is a positive interaction between cigarette smoking and PM_2.5_ for cardiovascular mortality [152]. It is likely that smoking or history of cigarette smoking may increase wildfire smoke-induced cardiovascular morbidity; however, to our knowledge, no studies have specifically investigated such an interaction. Nonetheless, many studies do consider smoking and smoking history as a co-variate on the association between wildfire smoke exposure and cardiovascular diseases [18, 118].

#### Limitations of epidemiological studies of the effects of wildfire smoke

As shown in Table 3, a number of studies showed positive association between wildfire smoke exposure and cardiovascular endpoints while several presented null associations. The inconsistency of the findings might result from differences in study design, endpoint choices, case count, and exposure assessment.

**Table 3:** Characteristics of the studies reviewed

Study type	Number of studies	Number of studies showing positive association	Major findings	Strength	Limitation
Epidemiological studies on wildfire smoke	48	33	Wildfire smoke exposure (most using PM_2.5_ or PM_10_ as exposure metric) is associated with increased morbidity and mortality of IHD, HF, CAD, CHD, and arrhythmia	Direct investigation on the cardiovascular effects of wildfire smoke exposure; relatively straightforward availability of hospitalization records and air quality (mainly PM) data	Exposure misclassification; limited causal inference; difficult to capture subclinical cardiovascular changes; failure to consider the effects of gases (O_3_, NO_2_, etc) and VOCs from wildfire smoke
Epidemiological studies on household biomass smoke	29	26	Indoor biomass smoke exposure is associated with increased blood pressure, vascular dysfunction, circulating vascular markers, and cardiovascular morbidity and mortality	Study sites and length are not restricted to wildfire events; straightforward follow-up with subjects using household fuels; applicability of interventions; can study subclinical endpoints	Household biomass smoke is not equal to wildfire smoke; not ideal for causal inference, cannot account for real-world wildfire events
Intervention / controlled human exposure studies	15	11	There are significant changes in vascular function, blood pressure, HRV, circulating cardiovascular and inflammatory markers	Controlled exposure and environmental conditions; potential for causal inference; detailed smoke characterization; randomization and cross-over design	Wood smoke does not equal wildfire smoke; cannot account for other environmental conditions in the real world wildfire events; exposure time is relatively short
In vivo animal studies	7	6	At different extent, wood /peat smoke condensate cause cardiovascular dysfunction, and increase inflammatory and cardiovascular injury markers	Detailed wood smoke characterization; controlled dose and exposure duration; sound model to examine cardiovascular function; sound for mechanistic studies	Wood smoke does not equal wildfire smoke; difficult to generalize the information to human exposure; intratracheal aspiration as exposure method in some studies is different from inhalation
In vitro studies	9	8	Wood smoke extract can induce cytotoxicity, oxidative stress and altered levels of inflammatory and vascular markers	Detailed wood smoke characterization; controlled dose and exposure duration; sound for mechanistic studies	Wood smoke does not equal wildfire smoke; difficult to generalize the information to human exposure

Some epidemiological studies employed an integrated approach to include large-scale healthcare and mortality data that may not be representative of the whole population. Some studies noted that null findings for cardiovascular effects were partly attributed to the low cardiovascular case counts, leading to wide confidence intervals, relative to respiratory case counts [8]. Outcomes based on healthcare needs for cardiovascular issues are likely to limit the investigations to certain susceptible subpopulations, while omitting healthier subgroups who may have developed small but significant pathophysiological effects, involving oxidative stress and inflammation.

Epidemiological studies are also prone to exposure misclassification due to their dependence on the use of exposure data from air quality monitoring stations or satellite images [85]. The broad approach applies exposure data based on smoky days, smoky area, scale of burnt forests, or air pollutant levels for the entire region [48, 54, 118], but does not consider individual-specific differences such as activity level, time spent indoors versus outdoors, and wind direction changes that may drastically affect the actual concentration of wildfire smoke. Total suspended particle levels from a study on biomass smoke derived from agricultural combustion of sugar cane, for example, may not specify particulate size information that is toxicologically relevant [16]. It has been shown that the amount of wood combustion in a city does not necessarily guarantee that the measured PM_10_ levels are representative of wood smoke exposure [44, 135].

Exposure misclassification can also be due to data quality factors or variations in the statistical methods used to process the data. Several studies surveyed in this review failed to distinguish between PM from wildfire smoke or background urban PM, while a few studies subtracted the background levels [10, 84, 85, 111]. In addition, fire intensity does not necessarily correspond to smoke levels and thus is not a sound metric for smoke exposure assessment [10]. The PM_2.5_ results based on modeled air pollution level seem to vary from model to model. Yao et al. [165] found that even though close in values, modeled PM_2.5_ concentrations, but not the measured levels, were associated with increased physician visits for cardiovascular disease on smoky days during the forest fire seasons of 2003 through 2010 in British Columbia, Canada. Gan and colleagues estimated PM_2.5_ concentrations based on both satellite and in situ data using three distinct methods: the Weather Research and Forecasting with Chemistry chemical-weather model, the Kriged interpolation of PM_2.5_ data from of in situ surface monitors, and a geographically weighted regression. The three models overall generated consistent PM_2.5_ exposure levels, but there were some significant differences among specific locations where the models were used, resulting in varying conclusions regarding the associations [53].

A majority (42 out of 48) of the wildfire smoke epidemiological studies reviewed (Table S1) utilized PM as an exposure metric for wildfire smoke in a variety of timelines and size fractions, and only 6 studies examined measured levels of gases (e.g. O_3_, CO) and volatile organic compounds (e.g. levoglucosan). Null findings between exposure to wildfire smoke and cardiovascular health effects may be due to the lack of accounting for gases, VOCs, PAHs and SOA, as well as cumulative exposure and co-exposures. While monitoring gaseous components from wildfire smoke can be challenging, some studies did propose indirect methodology including the environmental markers such as delta-C and metabolic markers such as 1-OHP for exposure assessment of PAHs from wildfire smoke [12, 13, 45]. However, such methods may not best represent exposure to organic compounds due to the low correlation between these markers and the actual air pollutant levels. For example, in one study the Pearson correlation factor between the delta-C levels and ambient PM_2.5_ levels was only *R* = 0.26 [18].

Some other limitations are identified in epidemiological studies that may lead to bias when interpreting results from these studies. For example, one study did not adequately account for important covariates such as temperature, humidity, and time trends [126]. Another study may have low power to detect an effect as it claims a positive finding with only a correlation between 2-day exposure and 2-day mortality data [157]. In addition, the choice of groups compared is questionable in this study, where frequency of pulmonary hypertension among women exposed to biomass smoke was higher than that among men who were cigarette smokers [141]. All the limitations discussed here may contribute the inconclusive results of a positive association between wildfire smoke exposure and cardiovascular effects in epidemiological studies.

### Controlled clinical exposure studies

Controlled clinical exposure studies complement the cardiovascular mortality and morbidity observations in epidemiology as they offer a chance to examine the associated pathophysiological changes. As shown in Table 3 and S3, wood smoke used in clinical and experimental studies in laboratory settings may not represent wildfire smoke entirely. In a clinical chamber setting, it is possible to maintain a relatively consistent wood smoke concentration, coupled with controllable environmental conditions such as temperature, humidity, and barometric pressure. In addition, chamber studies also allow researchers to characterize the chemical composition of the wood smoke (Table 1) and potentially investigate their roles in the cardiovascular effects. Low or moderate exercise can be introduced in the study protocols to increase the ventilatory rate to simulate physical activity in the vicinity of a wildland fire. The randomization and crossover design utilized in the chamber studies significantly reduce the influence of confounding covariates and are statistically efficient [25]. These considerations in clinical exposure studies significantly avoid exposure misclassification and provide much higher confidence in the causal inference of observed associations between wood smoke exposure and cardiovascular effects. These studies often purposely focus on the combustion of a single type or source of combustion material (e.g., red oak from a single tree or neighboring trees harvested from a single location) in order to minimize variability in the exposures from subject to subject.

Most clinical wood smoke exposure studies conducted to date have investigated pulmonary function, respiratory inflammation and systemic inflammatory responses, while about a dozen included cardiovascular endpoints or markers related to vascular pathophysiology. Commonly investigated cardiovascular endpoints include heart rate variability (HRV), vascular function, blood pressure, circulating endothelial adhesion molecules (e.g. ICAM1, VCAM1 and p-selectin), and coagulation proteins (vWF, tPA, and D-dimer). HRV is a reflection of the autonomic nervous system control on the heart and is quantified in the time domain with measurements of the time interval between consecutive heartbeats, and in the frequency domain, which measures the absolute or relative amount of signal energy within component bands [154]. An optimal level of HRV is associated with cardiac health; decreased HRV among healthy individuals is reported as a risk factor of cardiovascular morbidity and mortality [151]. Controlled chamber exposure to air pollutants, such as concentrated ambient fine and ultrafine PM, can significantly decrease HRV among middle-aged healthy subjects [150]. In one study, 14 subjects underwent a 3-h controlled exposure to a mean PM_1_ concentration at 314 μg/m^3^ of wood smoke generated through pyrolysis of birch wood with intermittent moderate exercise (an average minute ventilation of 20 L/min/m^2^ body surface area (BSA)). The authors found that HRV markers in the time domain, including standard deviation of successive NN intervals (SDNN), root-mean square of successive NN interval differences (RMSSD), and the proportion of NN50 divided by the total number of NN (R-R) intervals (pNN50), were significantly decreased 1-h after wood smoke exposure compared to filtered air [154]. In another study, 43 subjects participated in various training exercises that included activities to extinguish fires fueled by either wood alone or by wood and household materials (mattress). They found that firefighting activity was associated with decreased HRV and microvascular function measured as reactive hyperemia index [14]. On the other hand, a few studies have reported no significant changes in HRV following exposure to red oak wood smoke (485 ± 84 μg/m^3^) [57, 58], or to Danish beech wood smoke (13, 222, and 385 μg/m^3^) [24].

Several studies investigated changes in vascular function and blood pressure post exposure to wood smoke. Exposure to birch wood smoke significantly increased heart rate and central arterial stiffness, though not blood pressure [154]. After 1-h exposure to wood smoke measured as PM_1_ at about 1000 μg/m^3^ of birch wood smoke with intermittent exercise (average minute ventilation target 20 L/min/m^2^ BSA), 16 male firefighters showed significantly increased blood carboxyhemoglobin and increased vasodilatory response to bradykinin infusion compared with controls exposed to clean air, but no change in arterial pressure or pulse wave velocity [71]. The concentrations of the endothelial adhesion molecules ICAM1 and VCAM1 showed changes in the blood of healthy adults following a 3-h resting exposure to wood smoke corresponding to start-up and burn-out phases [144]. A few other studies failed to find any positive effects on vascular endpoints, but did report that blood markers related to vascular pathophysiology, such as coagulation factors, lipid peroxidation and oxidative stress, were significantly increased after wood smoke exposure [20, 50].

In a clinical study that included six different cookstoves that represent different combustion conditions, 48 subjects each underwent 2-h wood smoke exposures over six sessions at PM_2.5_ doses ranging from 0 to 500 μg/m^3^. Overall, the results showed that mean SBP, but not DBP, was 2 to 3 mmHg higher for all but one stove emission. All cookstove treatments also marginally increased pulse wave velocity and central augmentation index after 24 h compared to control. These results raised concerns that even low level PM_2.5_ from the most efficient cookstove can pose adverse cardiovascular effects [49, 158]. Similar reductions of cardiovascular effects associated with use of efficient energy usage to reduce indoor biomass smoke levels were also found in other observational studies [6, 34, 164].

### In vivo animal studies

As with clinical exposure studies, wood smoke used in the animal studies surveyed was derived from the combustion of one or two types of wood. The exposure methodology was either inhalation by animals breathing spontaneously in a small chamber, or intratracheal instillation of an extract prepared from condensed wood smoke. As summarized in Table S4, several studies have shown that exposure to wood smoke leads to increased cardiovascular risk in animals. Kim and colleagues established an in vivo model wherein female CD-1 mice were exposed to 100 μg peat smoke condensate from different combustion stages (smoldering vs. near-extinguished) through oropharyngeal aspiration. The mice exposed to the smoke produced during the smoldering-stage showed significantly decreased cardiac function and increased post-ischemic cardiac death in an experimentally induced ischemia model [82]. Another study demonstrated that Sprague-Dawley (SD) rats exposed to peat fire smoke extracts through oropharyngeal aspiration showed significantly higher end-systolic volume and pulmonary artery blood flow acceleration/ejection time ratios. Though minimal immune cell changes were observed in the bronchoalveolar lavage fluid (BALF), high doses of peat smoke PM extract (350 μg per animal) led to altered regulation of ventricular ejection and filling volumes, affecting blood flow in the pulmonary circulation [148]. In a study investigating the cardiometabolic responses to a high fat diet, rats exposed to peat fire smoke for 1 h showed increases in serum high-density lipoprotein (HDL) and total cholesterol, as well as increased isovolumic heart relaxation time after high fat food challenge, compared to values seen in control rats exposed to filtered air [93].

On the other hand, Reed and colleagues did not find any significant changes in cardiovascular markers among different animal strains (F344 rats, SHR rats, A/J mice, and C57BL/6 mice) after subchronic exposure to mixed oak wood smoke [122]. The animals in this study were whole-body exposed to different concentrations of wood smoke particulates (30, 100, 300, and 1000 μg/m^3^) for 6 h per day, 7 days a week for either 1 week or 6 months. The investigators evaluated cardiovascular function using electrocardiograms. No consistent changes were apparent under the wood smoke exposure conditions of this study, although increases in platelet numbers and decreases in blood urea nitrogen and serum alanine aminotransferase were reported [122]. Animal studies have also shown that the toxicity of wood smoke depends on the wood type as well as the combustion conditions, with wood smoke from either smoldering or flaming oak showing weaker effects relative to smoke produced by the burning of a few other wood types [81, 83, 140]. More details on how composition of wood smoke will affect its toxicity will be discussed in the next section.

## Mechanisms of cardiovascular impacts of wildfire smoke

As summarized in Table 3, in vivo animal and in vitro studies may often be challenging to interpret in a clinical context, as exposure dose and duration may be difficult to compare with real world conditions impacting public health. However, these studies provide insight into underlying pathophysiological mechanisms. In this section, we will focus on evidence from the literature including controlled human exposure studies, in vivo animal studies and in vitro studies to discuss the possible mechanisms of wildfire smoke induced cardiovascular effects.

**Fig. 2:**
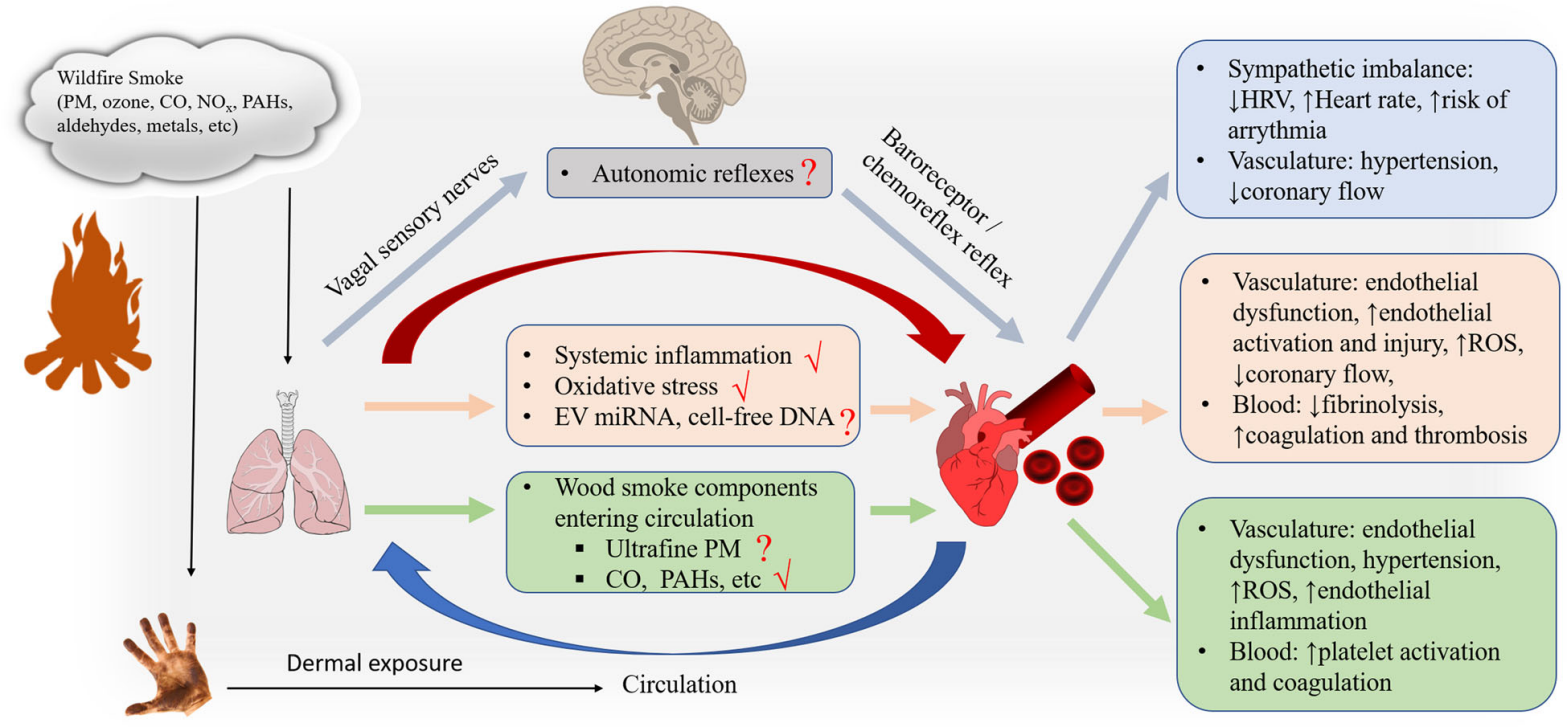
Schematic pathway showing proposed pathophysiological mechanisms of cardiovascular effects induced by wildfire smoke exposure. Wildfire smoke contains PM, gases, PAHs, and VOCs, etc. These components may affect the cardiovascular system through inhalational route, with a contribution from dermal exposure. Wildfire smoke can cause cardiovascular effects through three possible pathways: activation of autonomic reflex, oxidative stress and systemic inflammation, and direct interaction with the vasculature. Abbreviations: BP: blood pressure, CO: carbon monoxide, EV: extracellular vesicle, HRV: heart rate variability, miRNA: microRNAs, NO_x_: oxides of nitrogen, PAHs: polycyclic aromatic hydrocarbons, PM: particulate matter, ROS: reactive oxygen species

Several hypotheses have been proposed regarding the biological mechanisms underlying the health effects of air pollution. Among them are three principal pathways supported by evidence from epidemiological, clinical and toxicological studies [108, 146]. First, air pollutants can directly interact with neural receptors in the respiratory system and activate the autonomic nervous system, which has an impact on the heart rhythm and blood pressure. Second, pulmonary exposure to air pollutants produces oxidative stress and local and systemic inflammation, leading to lipid peroxidation, increased platelet activation and thrombosis, inflammation of vascular endothelia, and increased vasomotor tone. Lastly, small molecules of air pollutants, such as ultrafine PM and gases, may translocate through alveolar membranes and cause cardiovascular effects, including endothelial activation and injury [30, 99, 116]. There is limited research conducted directly on the mechanisms of cardiovascular effects caused by wildfire smoke, but after reviewing direct and indirect evidence from the literature, we postulate possible mechanistic pathways of cardiovascular effects induced by exposure to wildfire smoke based on these three notions (Fig. 2).

### Autonomic nervous system imbalance

As discussed earlier, wood smoke exposure under controlled conditions can significantly decrease HRV (i.e., SDNN, RMSSD, pNN50), a commonly examined endpoint of autonomic nervous system function [14, 154]. Inhalation of air pollutants, such as wildfire smoke, triggers autonomic reflexes in pulmonary receptors, baroreceptors, and chemoreceptors, leading to altered functional outcomes in the cardiovascular system [116].

Vagal sensory nerves and neural receptors in the respiratory system function to maintain normal respiratory physiology and pulmonary defense, and are sensitive to various environmental irritants [95]. These respiratory receptors include those found on unmyelinated C fibers (e.g., TRPA1, TRPV1, and J receptors), rapidly adapting pulmonary receptors (RAR), and slowly adapting pulmonary receptors (SAR) [116, 162]. Although not tested in wildfire smoke, studies of individual components present in wildfire smoke have shown potential to activate these receptors. For example, inhalation of high levels of ozone or volatile aldehydes can cause cardiovascular and pulmonary injury through activation of the TRPA1 receptor in humans and animals [28, 35, 62].

Activated sensory nerves send signals to the central nervous system, leading to modulation of the baroreceptor reflex that controls blood pressure and normal cardiovascular function [36]. In addition, these sensory nerves signal to the carotid body chemoreceptors located at the bifurcation of the carotid artery to maintain homeostasis of O_2_, CO_2_, pH, temperature and blood pressure [91]. Multiple studies have reported significant changes in blood pressure following exposure to wildfire smoke or wood smoke in humans in epidemiological and clinical studies [21, 32, 49, 167]. Wildfire smoke components, including PM, ozone, and SO_2_, have been shown to significantly decrease baroreflex sensitivity or affect the activity of chemoreceptors in the carotid body [116, 130]. Nevertheless, more research is needed to directly investigate the role of autonomic nerves in the control of the cardiovascular effects of wildfire smoke exposure.

### Oxidative stress and systemic inflammation

Oxidative stress is believed to be a key mechanism by which exposure to air pollution causes cardiovascular morbidity and mortality [97]. Components of wildfire smoke particles such as metals, are known to induce oxidative stress through redox and non-redox cycling mechanisms [57–59, 134].

Cultured human pulmonary artery endothelial cells exposed to 40 μg/ml of wood smoke condensate for 1–4 h showed increased production of reactive oxygen species (ROS), loss of mitochondrial membrane potential, increased expression of the mitochondrial apoptosis regulator Bcl-2-associated X protein (BAX), DNA fragmentation, and increased mRNA expression of superoxide dismutase 1 (SOD1) and heme oxygenase 1 (HMOX1) [89]. The antioxidant n-acetylcysteine was shown to reverse the oxidative stress state and restore depleted intracellular glutathione levels following wood smoke exposure [89]. Wood smoke particles from smoldering red oak also induced significant increases in ROS detected using the non-specific sensor 2′,7′ –dichlorofluorescein diacetate [57, 58]. Though Miousse et al. did not find any significant changes in ROS following biomass smoke exposure, mRNA expression of catalase and HMOX1 was significantly increased at 24 or 72 h compared to controls in an in vitro study [100].

Wood smoke-induced oxidative stress can cause a disruption of the cell cycle and cellular apoptosis. In RAW264.7 cells, birch wood smoke extract suspended in cell culture media at 15–300 μg/ml decreased cellular metabolism and increased the number of cells entering the subG1 phase of the cell cycle after 24-h exposure [106]. Similarly, after 40-h exposure to 40 μg/cm^2^ of wood smoke extract, THP-1 cells entered S/G2 phase of the cell cycle, indicating decreased proliferation [23]. Studies have also shown evidence of cytotoxicity from exposure to wood smoke by using lactate dehydrogenase (LDH) release assay [57, 58, 100, 106] or MTT assay [168], although the positive results were not present in all studies partly due to different wood type, cell line, and doses and exposure duration.

Inhaled particulates elicit inflammatory responses in the lung, and a sufficient particle dose, reactivity, or lack of clearance may lead to a “spill-over” of inflammatory responses into the blood causing systemic inflammation [139, 146]. Clinical wood smoke exposure studies have consistently found significantly elevated blood markers of inflammation, including interleukin 1 beta (IL-1β), IL-6, IL-8, C-C motif chemokine ligand 2 (CCL2), clara cell protein 16 (CC16), and tumor necrosis factor α (TNFα) [50, 57, 58, 63, 144, 145]. In vivo animal studies also offered insights on wood smoke exposure induced inflammatory responses locally and systemically. Exposure of BALB/c mice to an organic wood smoke extract from emissions derived from spruce combustion significantly increased inflammatory responses (COX-2, MPO) in both respiratory and cardiovascular systems [46]. Serum collected from C57BL/6 mice exposed to oak wood smoke (PM_2.5_ at 380 μg/m^3^) for 24 h was able to induce pro-inflammatory responses (IL-6 and CXCL1), and elevated expression of adhesion molecules (VCAM1 and ICAM1) in murine endothelial cells [15]. Wood smoke produced from smoldering fire generated higher levels of aerosolized endotoxin and potently induced cytokine levels, neutrophil influx, and intracellular ROS production in mouse lavage fluid relative to emissions produced at the near-extinguishing stage [82]. In vitro toxicological investigations have yielded similarly consistent inflammatory responses caused by exposure to wood smoke extract across different experimental cell lines, exposure time, and doses [23, 57, 58, 90, 106].

Recent research has pointed to extracellular vesicle (EV) microRNAs (miRNA) released by exposed cells into the circulation, which may facilitate cell-to-cell communications and play a role in air-pollution induced cardiovascular effects. Studies have shown that exposure to PM_2.5_ or ozone, two important components of wildfire smoke, was associated with an altered profile of miRNA targets such as miR-126, miR-19b, miR-150, miR-155, miR-191, and let-7a in human circulation [31, 128]. Many of these miRNA targets were associated with cardiovascular pathophysiology [129]. In terms of wildfire smoke, Ruiz-Vera and colleagues found that the expression of plasma miR-126 and miR-155 was significantly higher in women exposed to wood smoke compared to those using liquid petroleum gas (LPG), a cleaner form of fuel. In addition, there was a significant association between miR-126 and miR-155 expression levels and urinary 1-OHP concentrations, an indicator of PAH exposure [131].

### Distinguishing between the effects of particles and absorbed volatile components

Studies show that UFPs with a mean aerodynamic diameter < 100 nm may translocate into the blood circulation through the alveolar-capillary barrier, reaching the heart and peripheral blood vessels [52, 136]. A more recent study found that nano-sized particles could be detected in surgical specimens of the carotid artery of animals and in the blood and urine of healthy human volunteers following acute inhalation of the particles [98]. Ultrafine wildfire smoke PM might have the same potential to cross the epithelial barrier to enter the pulmonary circulation.

In addition, when wildfire smoke particles deposit in the alveolar region, the majority of their available PAH-load may rapidly detach from the particles, and transfer across the epithelial barrier and diffuse into the bloodstream in an unmetabolized state [56, 70]. Interestingly, an in vitro study has shown that wood smoke-induced proinflammatory responses and cytotoxicity were mainly determined by the organic fraction, rather than the washed particulates [23]. In addition to the particulates, gaseous components of wood smoke including SO_2_, ammonia, NO_x_, and CO were found to be important contributors to the pro-atherosclerotic vascular response to air pollution exposure in mice [140]. A series of studies testing the chemical components of wood smoke generated from different kinds of biomass (red oak, pocosin peat, ponderosa pine needles, lodgepole pine, eucalyptus) at different combustion stage (smoldering versus flaming) showed different acute pulmonary toxicity and mutagenicity in mice [81, 83].

## Research needs

A growing number of studies have implicated exposure to wildfire smoke as a risk factor for CVD. However, significant knowledge gaps regarding the association remain. Future epidemiological studies would benefit from improved exposure assessments and the implementation of more sensitive indicators of cardiovascular dysfunction. Similarly, the development of new methodologies could allow researchers to acquire individualized wildfire smoke exposure assessment, capturing more accurate exposure data [26, 27]. In addition to index air pollutants, future studies should also consider the impact of VOCs, which may be an important driver of wildfire smoke effects. Besides large-scale morbidity and mortality data, the identification of sensitive biomarkers should warrant information on the early onset of injury and on the underlying mechanisms of cardiovascular effects induced by wildfire smoke exposure. Since the direct evidence is lacking, more research is needed on how wildfire smoke exposure induces cardiovascular effects through the autonomic nervous system and directly impacts the vasculature. Novel mechanistic concepts including the role played by circulating miRNAs and cell-free DNA in the circulation on endothelial activation and injury should also be investigated.

There is also a geographical disparity in terms of the number of studies representing different regions. Among the wildlife smoke epidemiological studies presented in Table S1, 29 studies are from North America, 1 from South America, 9 from Australia, 3 from Southeast Asia, and 6 from Europe. However, there were no studies from Africa where Savanna fires are common and intensification of wildfire frequency and scale is projected for the future [11, 39]. More wildfire smoke studies should be carried out in these underrepresented regions where the public health system is more vulnerable, especially under the circumstances of global warming and increased wildland-urban interface.

Data should serve as the scientific backbone for policymaking in public health issues. As wildfire events are likely to occur more frequently in the foreseeable future, scientific efforts should be directing attention to the possible interventional approaches to reduce wildfire smoke induced health effects. Physical methods such as HEPA-equipped air purifiers, have showed promise in reducing exposure to wildfire smoke or wood smoke, leading to reduced inflammation and improved vascular function [7, 78]. A wildfire smoke forecast that triggers interventions to reduce personal exposure at lower PM_2.5_ threshold could be beneficial to reduce respiratory and cardiovascular health burdens [119]. In addition, comprehensive public health measures, such as Rule 4901, to reduce wood smoke exposure have been shown to lead to reductions in cardiovascular cases and IHD cases among those 65 years and older [166]. More research on the effectiveness of these strategies and applicability at a larger population scale is warranted to generate more robust data for informed public health decisions.

## Conclusion

This review finds that wildfire smoke contributes to high levels of air pollutants, including coarse and fine PM, gases, PAHs and VOCs, during wildfire events. Exposure to these pollutants, with most evidence derived from studies of wildfire smoke PM, is a risk factor for adverse cardiovascular effects, especially among susceptible populations, including the elderly, pregnant women, and those with low SES. The young and healthy may also develop biological responses including systemic inflammation and vascular activation. The limited understanding between exposure to the gaseous components of wildfire smoke and the cardiovascular effects warrants more research. We have also discussed the importance of more accurate exposure assessment and measurement of more sensitive cardiovascular endpoints in future epidemiological studies. Clinical and toxicological research have highlighted several possible mechanisms of wildfire smoke-induced cardiovascular effects including systemic inflammation and oxidative stress, autonomic nervous system imbalance, release of EV mediators into the circulation, and direct interaction of translocated smoke components into the circulation. As large-scale wildfire events will likely occur more frequently in the future, more research is needed to understand and intervene in the cardiovascular effects of wildfire. With science-guided policy decisions, we may diminish the adverse impacts of wildfires through reducing exposure levels, mitigating toxicity, and protecting the most vulnerable.

## Supplementary Information

**Additional file 1: Supplemental Table 1.** Summary of epidemiological studies on wildfire smoke exposure and cardiovascular effects. **Supplemental Table 2.** Summary of studies on indoor and ambient biomass smoke exposure and cardiovascular effects. **Supplemental Table 3.** Summary of intervention and controlled human exposure studies of wood smoke exposure and cardiovascular effects. **Supplemental Table 4.** Summary of in vivo animal studies on wood smoke exposure and cardiovascular effects. **Supplemental Table 5.** Summary of in vitro studies on wood smoke exposure and biological effects related to cardiovascular system.

## Data Availability

All data reviewed and described is either included in this manuscript or available online in the relevant publications.

## Abbreviations

BALF: Bronchial alveolar fluid
BAX: Bcl-2-associated X protein
BC: Black carbon
BSA: Body surface area
CCL2: C-C motif chemokine ligand 2
CC16: Clara cell protein 16
CHF: Congestive heart failure
CO: Carbon monoxide
COPD: Chronic obstructive pulmonary disease
COX-2: Cyclooxygenase-2
CVD: Cardiovascular disease
CXCL1: C-X-C motif chemokine ligand 1
DBP: Diastolic blood pressure
ED: Emergency department
EV: Extracellular vesicles
HDL: High-density lipoprotein
HMOX1: Heme oxygenase 1
HRV: Heart rate variability
ICAM1: Intercellular Adhesion Molecule 1
IL: Interleukin
IHD: Ischemic heart disease
LDH: Lactate Dehydrogenase
LPG: Liquid petroleum gas
MI: Myocardial infarction
MIP-2: Macrophage inflammatory protein 2
miRNA: MicroRNA
MMP9: Matrix metallopeptidase 9
MPO: Myeloperoxidase
NAC: N-acetylcysteine
NO: Nitric oxide
NO_2_: Nitrogen dioxide
PAHs: Polycyclic aromatic hydrocarbons
PM: Particulate matter
pNN50: Proportion of NN50 divided by the total number of NN (R-R) intervals
RAR: Rapidly adapting pulmonary receptors
RMSSD: Root-mean square of successive NN interval differences
ROS: Reactive oxygen species
SAR: Slowly adapting pulmonary receptors
SBP: Systolic blood pressure
SDNN: Standard deviation of successive NN intervals
SES: Socio-economic status
SO_2_: Sulfur dioxide
SOA: Secondary organic aerosol
SOD: Superoxide dismutase
TNFα: Tumor necrosis factor α
tPA: Tissue plasminogen activator
TRPA1: Transient receptor potential ankyrin 1
TRPV1: Transient receptor potential vanilloid 1
UFPs: Ultrafine particles (< 100 nm diameter)
VCAM1: Vascular Cell Adhesion Molecule 1
VOCs: Volatile organic compounds
vWF: Von Willebrand factor
1-OHP: 1-Hydroxypyrene
