# Supplementary material for: Cardiovascular health impacts of wildfire smoke exposure
Source: Part Fibre Toxicol. 2021 Jan 7;18:2. doi: 10.1186/s12989-020-00394-8 (PMC7791832; doi:10.1186/s12989-020-00394-8)
Supplement: Supplementary file 1 — Additional file 1: Supplemental Table 1. Summary of epidemiological studies on wildfire smoke exposure and cardiovascular effects. Supplemental Table 2. Summary of studies on indoor and ambient biomass smoke exposure and cardiovascular effects. Supplemental Table 3. Summary of intervention and controlled human exposure studies of wood smoke exposure and cardiovascular effects. Supplemental Table 4. Summary of in vivo animal studies on wood smoke exposure and cardiovascular effects. Supplemental Table 5. Summary of in vitro studies on wood smoke exposure and biological effects related to cardiovascular system. [file 12989_2020_394_MOESM1_ESM.docx]

**Cardiovascular Health Impacts of Wildfire Smoke Exposure**

Hao Chen ^1*^, James M. Samet ^2^, Philip A. Bromberg ^3^, Haiyan Tong ^2^*

^1^ Oak Ridge Institute for Science and Education, Oak Ridge, TN 37830, USA.

^2^ Public Health and Integrated Toxicology Division, Center for Public Health and Environmental Assessment, U.S. Environmental Protection Agency, Chapel Hill, NC 27514, USA.

^3^ Center for Environmental Medicine, Asthma and Lung Biology, University of North Carolina at Chapel Hill, Chapel Hill, NC 27514, USA

* Corresponding authors: Haiyan Tong, MD, PhD, [tong.haiyan@epa.gov](mailto:tong.haiyan@epa.gov); Hao Chen, PhD, [chen.hao@epa.gov](mailto:chen.hao@epa.gov)

**Supplemental Table 1.** Summary of epidemiological studies on wildfire smoke exposure and cardiovascular effects.

| **Author & year** | | **Study type** | | **Smoke source** | **Exposure level** | | **Pulmonary effects** | | **Cardiovascular effects** | | | **Other** |
| --- | --- | --- | --- | --- | --- | --- | --- | --- | --- | --- | --- | --- |
| **Cardiovascular morbidity** | | | | | | | | | | | | |
| (Mott et al. 2005) | | Ecological time-series study | | 1997 forest fires in Southeast Asian | Smoky periods vs. non-smoky periods | | There is a significant association between fire-related smoke and respiratory hospitalizations, especially among COPD and Asthma patients | | Persons older than 65 years old are more likely to be re-hospitalized from post-fire period than from non-fire period due to cardiorespiratory disease | | | NA |
| (Moore et al. 2006) | | Ecological time-series study | | 2003 forest fire in British Columbia, Canada | PM_10_ and PM_2.5_ peaked at 200 and 250 µg/m^3^ respectively 6 days after the fire began | | Physician visits for respiratory diseases were significantly increased during the forest fire period in one region | | Forest fire effects on the physician visits for cardiovascular diseases were not found | | | NA |
| (Johnston et al. 2007) | | Case-crossover study | | 2000, 2004, and 2005 biomass/ vegetation fires in Darwin, Australia | PM_10_ ranged from 6.4 to 70 µg/m^3^ from April 1^st^ to November 30^th^ in 2002, 2004, and 2005 | | PM_10_ levels were significantly associated with increased admissions for all respiratory conditions with a larger magnitude among indigenous groups | | PM_10_ levels were not significantly associated with admissions for cardiovascular admissions overall, but with ischemic heart diseases among indigenous groups | | | NA |
| (Hanigan, Johnston, and Morgan 2008) | | Ecological time-series study | | Fire seasons in Darwin, Australia from 1996 to 2005 | PM_10_ peaked to 31.12 µg/m^3^ from April to June in 2005 | | Similar to the study above, increase of PM_10_ is associated with increase in total respiratory admissions | | PM_10_ increase was negatively associated with cardiovascular admissions on the same day of exposure, but positively 2-3 days later among indigenous people, though not significant | | | NA |
| (Delfino et al. 2009) | | Ecological time-series study | | 2003 wildfires in Southern California (October) | PM_2.5_ increased 70 µg/m^3^ during wildfire smoke compared to that of pre-wildfire period | | 2-day average of PM_2.5_ is associated with increased respiratory illness and asthma hospital admission during the fire. Certain age group (<65 yrs. old) are more susceptible | | Hospital admissions for cardiovascular illnesses were not associated with wildfire smoke | | | NA |
| (Henderson et al. 2009) | | Population-based cohort | | 2003 forest fire in British Columbia, Canada | PM_10_ was 29 ± 31 μg/m^3^, with an interquartile range of 14–31 μg/m^3^ | | A 30 μg/m^3^-increase in tapered element oscillating microbalance (TEOM) based PM_10_ is associated with increased physician visits for all respiratory illness, for asthma, and for respiratory hospitalizations | | Physician visits and hospital admissions for cardiovascular illnesses were not associated with PM_10_ exposure during wildfire period | | | NA |
| (Lee et al. 2009) | | Ecological time-series study | | 1999 forest fire near the Hoopa Valley Indian Reservation, California, USA | The comparison year was 1998 when there was no fire. Maximum PM_10_ is much higher in 1999 compared with 1998 (996.3 μg/m^3^ vs. 175 μg/m^3^). There were no days in 1998 that exceeded 150 μg/m^3^ for a 24-h average concentration, the national air quality standard for PM_10_ at the time, but 12 days in 1999 during fire period | | Daily PM_10_ levels during the wildfire were significant predictors for patients seeking care for asthma | | Daily PM_10_ levels during the wildfire were significant predictors for patients seeking care for coronary artery disease and headache. In addition, daily PM_10_ levels during the wildfire were significant predictors for patients seeking care for circulatory illness among residents of nearby communities and new patients | | | NA |
| (Morgan et al. 2010) | | Ecological time-series study | | Bush fire in Sydney, Australia from 1994 to 2002 | PM_10_ ranged from 3 to 117 μg/m^3^. Ozone ranged from 3 to 132 ppb | | A 10 μg/m^3^ increase in PM_10_ due to wildfire was associated with a 1.24% increase in all respiratory admissions at lag 0, a 3.80% increase in COPD admissions at lag 2 | | Cardiovascular disease admissions were only associated with a 10 μg/m^3^ increase in urban PM_10_, but not bush fire linked PM_10_ | | | NA |
| (Schranz, Castillo, and Vilke 2010) | | Ecological time-series study | | 2007 San Diego wildfires in California, USA | PM_2.5_ peaked at 80 μg/m^3^ | | The admission rate was higher in the period following the fires (19.8% vs. 15.2%) from the baseline period with major complaints of shortness of breath | | Patients with significant cardiac or pulmonary history were no more likely to present to the emergency department during the fires | | | NA |
| (Henderson et al. 2011) | | Population-based cohort | | 2003 forest fire in British Columbia, Canada | Three metrics for PM_10_: TEOM (avg. 29 μg/m^3^), CALPUFF avg 11.4 μg/m^3^), SMOKE (45.9 and 44.2 μg/m^3^) | | Similar to the study in 2009, a 30 μg/m^3^ increase in TEOM-based PM_10_ was associated with increased physician visits for all respiratory illness, for asthma, and for respiratory hospitalizations | | Associations with cardiovascular outcomes were largely null. A new aspect of this study indicated that exposure measurement tools other than air quality monitoring, such as satellite imaging data, might be useful | | | NA |
| (Rappold et al. 2011) | | Population-based cohort | | 2008 peat bog wildfire in North Carolina, USA | Comparisons between counties with and without the peat fire smoke | | Increased ED visits due to respiratory illness (RR: asthma 1.65, COPD 1.73, pneumonia and bronchitis 1.59) in counties exposed to the smoke | | Increased ED visits due to heart-failure related illness (RR: cardiopulmonary 1.23, heart failure 1.37) in counties exposed to the smoke, especially in the older population | | | NA |
| (Crabbe 2012) | | Ecological time series | | Bushfire in Darwin, Australia during 1993-1998 | PM_10_ (peak: 42.5 μg/m^3^), coarse PM (peak: 32.3 μg/m^3^), fine PM (peak: 20.7 μg/m^3^) and black carbon (peak: 3385 μg/m^3^) | | Respiratory admissions were associated with exposure to PM_10_ with a lag of 1 day, FPM, and black carbon | | Cardiovascular admissions had the strongest association with exposure to same-day PM_10_ and highest relative risk (RR) for exposure to FPM when adjusted for confounders | | |  |
| (Rappold et al. 2012) | | Population-based cohort | | Wildfire smoke in North Carolina, USA from June 1^st^ to June 14^th^, 2008 | PM_2.5_ ranged from 4 to 129 µg/m^3^ | | For asthma, the strongest association was observed at lag day 0 with excess relative risk of 66% per 100 µg/m^3^ increase in PM_2.5_ | | For congestive heart failure, the excess relative risk was 42% per 100 µg/m^3^ increase in PM_2.5_ | | | Socioeconomic status is an effect modifier of the association |
| (Hejl et al. 2013) | | Crossover timeseries study | | Wood smoke from prescribed fire with fuels consisted of pine, mixed hardwood and pine, etc., in Savannah River, South Carolina in 2011 | The unadjusted PM_2.5_ geometric mean was 650 μg/m^3^ ranging from 288 to 1306 μg/m^3^, while the unadjusted CO geometric mean was 3.6 ppm ranging from 1.54 to 19.85 ppm. | | NA | | Cardiovascular markers in blood, including SAA, ICAM-1, VCAM-1 increased >50% of samples across the shift | | | A significant cross-work shift increase in IL-8 in blood samples was observed in healthy wildland firefighters working at prescribed burns |
| (Martin et al. 2013) | | Ecological time series | | Bushfires in three eastern Australia cities from 1994 to 2007. Sydney 58 days,  Wollongong 33 days,  Newcastle 50 days | Sydney (PM_2.5_, PM_10_: 2-100, 1.6-199 µg/m^3^),  Wollongong (PM_2.5_, PM_10_: 1.6-112, 2.5-281 µg/m^3^),  Newcastle (PM_2.5_, PM_10_: 1.8-62, 2-161 µg/m^3^) | | Smoke events were associated with increased hospital admissions for the same-day respiratory illnesses in all three cities | | Smoke events were not associated with increased hospital admissions for the same-day cardiovascular illnesses | | | NA |
| (Gaughan et al. 2014) | | Cross-sectional study | | Two crews of wildland firefighters in Colorado | Urinary levoglucosan levels was used as an indicator of biomass burning exposure | | NA | | Mean augmentation index % was higher for participants with higher oxidative stress scores. The association remained significant after adjusting for smoking status | | | Higher levoglucosan concentration was positively associated with oxidative stress. |
| (Rappold et al. 2014) | | Simulated forecast-based intervention case study | | 2008 Evans Road fire in North Carolina | Out of 1364 county-days, 238 smoke related PM_2.5_ > 5 μg/m^3^, 107 had > 20 μg/m^3^, and 37 had > 50 μg/m^3^ | | Per 10 μg/m^3^ increase of PM_2.5_, smoke exposure from wildfire was associated with an excess RR for asthma-related ED visits | | Per 10 μg/m^3^ increase of PM_2.5_, smoke exposure from wildfire was associated with an excess RR for heart failure-related ED visits | | | Forecast-based intervention analysis showed that triggering interventions at lower PM_2.5_ threshold is effective in reducing health risks and reduce health burden |
| (Johnston et al. 2014) | | Time-stratified case-crossover | | Forest fires in Sydney, Australia from 1996 to 2007 | Avg. PM_10_ (Non-smoke day: 17.8, smoke day: 60.5 µg/m^3^).  Avg. PM_2.5_ (Non-smoke day: 9.9, smoke day: 39.1 µg/m^3^) | | Smoke days were associated with increases in ED visits for all non-trauma conditions, respiratory conditions, COPD, and asthma. Positive effects persisted for 1-3 days post exposure | | Ischemic heart disease ED visits were increased at a lag of 2 days while arrhythmias had an inverse association at a lag of two days. A further association between smoke event and heart failure attendances was present for the 15–65-year age group | | | NA |
| (Le et al. 2014) | | Ecological study | | 2002 forest fires in Quebec, Canada. Population in U.S. states. | The avg. countywide PM_2.5_ was 53 µg/m^3^ during the haze days and 21.5 µg/m^3^ on the non-haze days | | The increased PM_2.5_ due to wildfire smoke is associated with increased hospitalizations for respiratory illnesses compared to that before the fume arrived | | The increased PM_2.5_ due to wildfire smoke is associated with increased hospitalizations for cardiovascular illnesses compared to that before the fume arrived | | | PM from wildfire can transport and impact health of elderly populations in a long distance |
| (Dennekamp et al. 2015) | | Ecological time-series (case crossover) | | Forest fire in Victoria, Australia from Nov. 2006 to Mar. 2007 | PM_2.5_ peaked at 247.2 µg/m^3^. In the 12 days with active fire, avg. PM_2.5_ was at 106 µg/m^3^ | | NA | | Among men, increase of out of hospital cardiac arrest cases was associated with PM_2.5_, PM_10_, and CO with 48-h lag during fire season. PM_2.5_ is the main contributor of the effects | | | NA |
| (Haikerwal et al. 2015) | | Ecological time-series (case crossover) | | Forest fire in Victoria, Australia from Dec. 2006 to Jan. 2007 | PM_2.5_, mean 15.43 µg/m^3^ and peak 163.44 µg/m^3^ | | NA | | An increase in interquartile range (IQR) of 9.04 µg/m^3^ in PM_2.5_ over 2-d moving average was associated with a 6.98% increase in risk of out-of-hospital cardiac arrests, with strong association shown by men and by older adults.  There is also an increased risk for IHD-related ED visits and IHD-related hospitalizations, especially among women and the elderly | | | NA |
| (Resnick et al. 2015) | | Ecological time-series | | 2011 Wallow fire in Albuquerque, New Mexico, USA | During acute exposure period, PM_2.5_, mean 31.3 and range 10-70 µg/m^3^ | | During heavy smoke conditions, there was a significant increase in ED visits among >65 group for asthma | | During heavy smoke conditions, there was a significant increase in ED visits among 65+ group for diseases of veins, lymphatic and circulatory system. Among the group of 20-64 years old, acute wildfire smoke was associated with increased ED visits for pulmonary circulation and cerebrovascular diseases | | | NA |
| (Alman et al. 2016) | | Ecological time series | | 2012 wildfires in Colorado, USA | 1-h average of PM_2.5_ range 2.02-5,000 µg/m^3^ during the wildfire season | | Increases in PM_2.5_ (either 5 or 10 µg/m^3^) was associated with asthma, COPD for lag 0, 2, and 3 days | | Cardiovascular results were consistent with no association although there were some wide confidence intervals due to low case counts for cardiovascular outcomes in ED visits | | | NA |
| (Ostro et al. 2016) | | Ecological time series (case crossover) | | Wildfire smoke in California, USA from 2005-2009 | Biomass burning contributes 2.6 out of the 16.4 µg/m^3^ avg. PM_2.5_. PMF model for source apportionment | | PM_2.5_ mass from biomass burning is significantly associated with increased ED visits for all respiratory illnesses and with increased asthma visits | | PM_2.5_ mass from biomass burning is not significantly associated with increased ED visits for all cardiovascular illnesses but only with the increased dysrhythmia visits at 2 days lag | | | NA |
| (Reid et al. 2016) | | Ecological time series | | 2008 northern California wildfire | 24-h avg. of PM_2.5_ 19.14 µg/m^3^ and avg. O_3_ 59.69 ppb during fire days | | During fires, PM_2.5_ was positively associated with asthma hospitalization and ED visits, and with increased ED visits for COPD | | During fires, null results were found between PM_2.5_ levels and cardiovascular disease outcomes | | | Age, gender and SES are modifiers for the association between ED visits for respiratory disease outcomes and wildfire smoke exposure |
| (Tinling et al. 2016) | | Population-based cohort | | 2011 Plains Bay wildfire in North Carolina, USA | During peak exposure, 24-avg. PM_2.5_ could reach 121.4 µg/m^3^ | | RR associated with a 10 μg/m^3^ increase in 24-h PM_2.5_ was significantly elevated in adults and youth for respiratory symptoms and upper respiratory infection | | RR associated with a 10 μg/m^3^ increase in 24-h PM_2.5_ was significantly elevated in adults for hypertension, and in youth for all cause cardiac outcomes | | | NA |
| (Yao, Eyamie, and Henderson 2016) | | Population-based cohort | | Forest fire seasons of 2003 through 2010 in British Columbia, Canada | Daily average PM_2.5_ during extreme fire days were measured 10.2 µg/m^3^ compared with 5.9 µg/m^3^ for all days; modeled 10.3 µg/m^3^ compared with 6.0 µg/m^3^ for all days | | Both measured and modeled PM_2.5_ were associated with increased physician visits for asthma, lower respiratory infections, and otitis media on extreme fire days, and not associated with increased visits for upper respiratory infections | | Only modeled PM_2.5_ were associated with increased physician visits cardiovascular disease on fire days | | | NA |
| (Gan et al. 2017) | | Time-stratified case crossover | | 2012 wildfire in Washington, USA | Smoke day was defined as any days with PM_2.5_ >= 10 µg/m^3^. Levels of PM_2.5_ levels were estimated using both *in situ* and satellite data | | A 10 μg/m^3^ increase in 24-h PM_2.5_ was associated with increased hospital admissions for asthma. However, the effects were not always significantly with all exposure levels across the three different models | | Overall cardiovascular hospital admissions were not associated with the PM_2.5_ levels, independent of the model types. But the associations were significant in cerebrovascular disease hospitalizations and among males | | | Estimates of PM_2.5_ for the same spatial location were based on satellite and in situ measures |
| (Garcia-Olivé et al. 2017) | | Ecological time-series | | Forest fires in Catalonia, Spain | Burnt forest surface area (%) | | The burnt forest area was not associated with increased hospitalizations | | The burnt forest area was not associated with increased cardiovascular hospitalizations | | | Study design has some issues |
| (Liu et al. 2017) | | Ecological study | | Wildfires in Western USA, 2004-2009 | Smoke wave standards were set for 23, 28, and 37 µg/m^3^ | | A 7.2% increase in risk of respiratory admissions during smoke wave days with high wildfire-specific PM_2.5_ (>37µg/m^3^) compared to matched non-smoke-wave days | | There was no association between smoke wave days and increased cardiovascular hospitalizations | | | NA |
| (Parthum, Pindilli, and Hogan 2017) | | Ecological study | | Peat fire in the Great Swamp National Wildfire Refuge in Virginia, USA | Smoke days vs. non-fire days | | The wildfire event significantly contributes to 35 excess ED visits for COPD, 20 for asthma, and 41 for pneumonia. | | The wildfire event significantly contributes to 32 excess ED visits for chronic heart failure and 33 visits for cardiopulmonary symptoms. | | | NA |
| (Salimi et al. 2017) | | Ecological time-series | | Wildfires in the Sydney metropolitan area in Australia, 2004-2015 | Forest fire smoke level of avg. PM_2.5_ were 16.4 µg/m^3^ | | A 10 μg/m^3^ increase in PM_2.5_ were positively associated with same day emergency ambulance dispatches (EAD) for respiratory problems and breathing problems | | Increases of 10 μg/m^3^ in PM_2.5_ were positively associated with same day EAD for cardiac arrest and chest pain on the same day and heart problems with a 2-day lag | | | NA |
| (Weichenthal et al. 2017) | Time series (case crossover) | | Wildfire smoke in British Columbia, Canada, 2008-2015 | | | Daily avg. PM_2.5_ was 8.8 (SD: 7.4) μg/m^3^. Levoglucosan level was also measured to confirm if the source of PM_2.5_ was from wood smoke. There is a good correlation between PM_2.5_ and levoglucosan | NA | | Each 5 µg/m^3^ increase in 3-day mean PM_2.5_ was associated with an increased risk of MI among elderly subjects (≥65 yrs. old), but not among younger people. In colder periods, the association was stronger due to biomass burning | NA | | |
| (Wettstein et al. 2018) | | Population-based study | | 2015 wildfire smoke in California, USA | Smoke density level: light (PM_2.5_ 0–10 µg/m^3^), medium (10.5– 21.5 µg/m^3^), and dense smoke (22+ µg/m^3^) | | Respiratory conditions were increased with the increases in smoke density | | Elevated risks for individual diagnosis including myocardial infarction, ischemic heart disease, heart failure, ischemic stroke, pulmonary embolism, etc, were observed with increased smoke density | | | The observed risk was greatest among adults aged >65 years |
| (Abdo et al. 2019) | | Ecological | | Wildfires in Colorado, USA, 2007-2015 | The trimester-average wildfire smoke of PM_2.5_ was low with a mean of 0.2 and max of 4.5 µg/m^3^ | | NA | | A 1 µg/m^3^ increase in PM_2.5_ exposure to wildfire smoke over the full gestation and during the 1^st^ and 2^nd^ trimester was positively associated with gestational hypertension | | | Increased PM_2.5_ levels were also associated with pre-term birth and low birth weight |
| (Deflorio-Barker et al. 2019) | | Ecological time-series | | Wildfires in the USA, 2008-2010 | Smoke days vs non-smoke days, PM_2.5_ was significantly higher during smoke days | | For asthma-related hospitalizations, there is a higher risk during smoke days than that of non-smoke days | | The increased risks of PM_2.5_ related cardiopulmonary hospitalizations were similar between smoke and non-smoke days | | | NA |
| (Stowell et al. 2019) | | Case-crossover design | | Wildfire in Colorado, USA 2011-2014 | Ground measurements, chemical transport models, and remote sensing data were combined | | A 1 μg/m^3^ increase in fire smoke PM_2.5_ was associated with increased risk for asthma (OR = 1.081 (1.058, 1.105)) and combined respiratory disease (OR = 1.021 (1.012, 1.031)) | | No significant relationships were evident for cardiovascular diseases and the smoke PM_2.5_ | | | NA |
| (Jones et al. 2020) | | Case-crossover study | | Wildfires in California, USA between 2015-2017 | Exposure levels were categorized into light, medium, and heavy based on smoke density using satellite imaging | | NA | | Out-of-hospital cardiac arrest risk increased in association with heavy smoke across multiple lag days, strongest on lag day 2 (odds ratio, 1.70; 95% CI, 1.18–2.13). Low SES may increase the risk | | | Higher risk among people who are 35 years and older on days with heavy smoke |
| **Cardiovascular mortality** | | | | | | | | | | | | |
| (Vedal and Dutton 2006) | | Ecological study | | 2002 wildfire in Denver, Colorado, USA (June) | PM_10_ and PM_2.5_ peaked at 372 and 200 µg/m^3^ respectively 6 days after the fire began | | Small increase in daily mortality for respiratory diseases cannot be attributed to wildfire smoke | | Small increase in daily mortality for cardiovascular diseases cannot be attributed to wildfire smoke | | | NA |
| (Analitis, Georgiadis, and Katsouyanni 2012) | | Ecological time series | | Forest fires in Athens, Greece during 1998-2004 | Black smoke concentration (during large forest fire: 36.4$\pm$9.7 μg/m^3^) | | All mortality and respiratory mortality were associated with large fires | | Dose response cardiovascular mortality was observed with large fires. The effects were larger among those aged < 75 years old | | | NA |
| (Marlier et al. 2013) | | Ecological study | | Satellite-derived fire estimates and atmospheric modelling in Southeast Asia from 1997 to 2006 | During strong El Niño years, fires contribute up to 200 µg/m^3^ and 50 ppb in annual avg. PM_2.5_ and O_3_ levels near fire | | NA | | Wildfire contributes to 200 additional days per year that exceed the WHO 50 µg/m^3^ 24-hr PM_2.5_ interim target and an estimated 10,800 (6,800–14,300)-person (about 2%) annual increase in regional adult cardiovascular mortality | | | NA |
| (Nunes, Ignotti, and Hacon 2013) | | Ecological study | | 2005 biomass fires in Brazilian Amazon | Annual percentage of hours of PM_2.5_ > 25 µg/m^3^ | | NA | | Compared with low PM_2.5_ exposure hours, high exposure leads to significant increase of cardiovascular mortality at all age groups, acute MI among groups >75 years old | | | NA |
| (Shaposhnikov et al. 2014) | | Ecological time-series | | 2010 wildfire in Moscow, Russian | June 1^st^ to Aug. 31^st^, 2010, the 24-h avg. of PM_10_ reached as high as 300 µg/m^3^. | | All non-accidental mortality and respiratory mortality increased during the wildfire season | | Cardiovascular mortality increased during the heat wave and wildfire season | | | NA |
| (Linares et al. 2015) | Ecological time series | | Biomass combustion in Madrid, Spain, 2004-2009 | | | Biomass smoke days were defined. 56 out of 2192 days had biomass combustion. PM_2.5_, PM_10_ and O_3_ on days with advection (PM_2.5_, 23.6 vs. 16.9; PM_10_, 44.2 vs. 31.1; O_3_, 48.0 vs. 36.4) | | PM_10_ had greater impact on the organic mortality with advection than did PM_2.5_ without advection. Among people 75 yrs. older, PM_10_ was associated with respiratory mortality | No association between cardiovascular mortality and particulate matter exposure was found | | NA | |
| (Faustini et al. 2015) | | Ecological time-series (case crossover) | | Forest fires in Mediterranean area from 2003 to 2010 | Satellite data were used for exposure assessment. Smoky day was defined as a day when PM_10_ was higher than 8 µg/m^3^ | | A total of 391 smoky days in all cities. Smoky days were associated with increased all-natural and respiratory mortality which was also much higher than that of non-smoke days | | Smoky days were associated with increased cardiovascular mortality at lag 0–5 | | | NA |
| (Kollanus et al. 2016) | | Ecological time series (case crossover) | | Vegetation fire smoke that affect Helsinki, Finland during 2001-2010 | 24-h avg. of PM_2.5_ 30 µg/m^3^, PM_2.5-30_ 18 µg/m^3^, and 8-h avg. O_3_ 89.7 µg/m^3^ during days | | A positive association between smoke day PM_2.5_ and hospital admissions due to respiratory causes among the elderly | | 10 µg/m^3^ increase in PM_2.5_ was associated with all cardiovascular mortality with 3-day lag, and also among 65+ population with the same day effect and 3-day lag. Hospitalization of cardiovascular diseases were not associated with smoke days | | | Long-distance of air pollution translocation |
| (Navarro et al. 2019) | | Modeling and estimate study | | Firefighter conducting firefighting activities during wildfire events in the USA | A mean concentration 0.51 mg/m^3^ of PM_4_ due to smoke exposure (49 days per year) | | It was estimated that wildland firefighters were at an increased risk of lung cancer (8 percent to 43 percent) | | Wildland firefighters were also at an increased risk of CVD (16 percent to 30 percent) mortality | | | NA |
| (Uda, Hein, and Atmoko 2019) | | Ecological | | Peatland fires in Indonesia, 2011-2015 | An avg. increase in the annual mean PM_2.5_ due to peatland fire was 26 μg/m^3^ | | Excessive mortality due to respiratory diseases were correlated with peatland fire smoke | | Excessive mortality due to cardiovascular diseases were correlated with peatland fire smoke | | | NA |

**Supplemental Table 2**. Summary of studies on indoor and ambient biomass smoke exposure and cardiovascular effects.

| **Author & year** | | **Study type** | **Fuel type** | **Exposure level** | **Pulmonary effects** | | **Cardiovascular effects** | **Other biological markers** | |
| --- | --- | --- | --- | --- | --- | --- | --- | --- | --- |
| **Indoor biomass smoke** | | | | | | | | | |
| (Baumgartner et al. 2011) | | Panel study (repeated measures) | Household biomass combustion in Yunnan, China | 24-h avg. PM_2.5_ levels range from 22 to 634 µg/m^3^ | NA | | A 1‑log‑µg/m^3^ increase in PM_2.5_ exposure was associated with 2.2 mm Hg higher systolic blood pressure (SBP) and 0.5 mm Hg higher Diastolic BP (DBP) among all women > 50 yrs. old. Not significant among young women | NA | |
| (Clark et al. 2011) | | Cross-sectional study | Household biomass combustion in Granada, Nicaragua | 48-h avg. PM_2.5_ levels 154 - 6901 µg/m^3^, 48-h indoor CO (0.4-123.82 ppm), and personal CO (0.07-14.08 ppm) | Per IQR increase of PM_2.5_ or CO, there was no significant changes in lung function test (FEV_1_ and PEF) | | SBP was not significantly changed due to increase of CO, but the relationship was prominent among obese groups. No significant association between PM_2.5_ and SBP | NA | |
| (Kargin et al. 2011) | | Cross sectional (46 exposed, 31 control) | Household combustion of wood and other types of biomass | NA | Biomass fuel exposure caused obstructive and restrictive spirometry impairments | | Biomass fuel exposure caused both systolic and diastolic left ventricular (LV) function impairment in patient exposed to biomass fuel | NA | |
| (Clark et al. 2013) | | Interventional panel study. Baseline is from Clark 2011 | Household biomass combustion in improved cookstove in Granada, Nicaragua | Mean 48-h were reduced from 1801 μg/m^3^ to 416 μg/m^3^ for PM_2.5_, 25.8 ppm to 7.2 ppm for indoor CO, and 2.1 ppm to 0.8 ppm for personal CO | NA | | Substantial reductions in blood pressure were not observed among the entire population with improved cookstove, but the reductions were significant among women 40+ yrs old in SBP and among obese subjects | NA | |
| (Painschab et al. 2013) | | Cross sectional study | Biomass combustion in Puno, Peru | Median 24-h concentrations of indoor PM_2.5_ were 14 μg/m^3^ in the clean fuel homes versus 280 μg/m^3^ in the biomass fuel homes | NA | | The biomass fuel group has higher levels of carotid intima media thickness (CIMT), carotid plaque prevalence, and SBP compared with that of clean fuel groups | NA | |
| (Shan et al. 2014) | | Cross sectional study | Wood combustion for cooking 2-3 times a day in rural China | Geometric 24-h mean of PM_2.5_ were 61 μg/m^3^ overall, 101 and 39 μg/m^3^ in the high and low exposure group. 58 μg/m^3^ was the cutoff point for high and low groups | NA | | Women in high exposure group showed higher trend of systolic SBP, central SBP, central pulse pressure, and augmentation index than those of low-exposure group, though not statistically significant | Telomere was marginally shorter among high-exposure group. The CRP levels were not different from the two groups | |
| (Alexander et al. 2015) | | A panel study on before and after the intervention | Primary fuel source were hardwood and shrubs in Tuquiza, Bolivia | Kitchen PM_2.5_ pre-intervention levels ranged 23-750 (avg. 240) μg/m^3^, post levels range 8-170 (avg. 48) μg/m^3^ | NA | | Mean SBP were decreased from 114.5 to 109.0 mm Hg after the intervention. There were also stronger association between SBP reductions and 24-h kitchen PM as well as cooking PM concentration | NA | |
| (Ruiz-Vera et al. 2015) | | Cross sectional study | Indoor biomass combustion for cooking in San Luis Potosi, Mexico | 1-hydroxypyrene (1-OHP) was the biomarker of PAH exposure. The urinary 1-OHP levels in the study was 11 times higher than the mean reported in general population | NA | | Vascular function measured by high resolution ultrasonography was significantly associated with urinary 1-OHP levels | NA | |
| (Wylie et al. 2015) | | Cross sectional study | Indoor wood combustion in central East India | Wood combustion group vs. gas fuel group (gas was considered as a clean energy type) | NA | | Compared to gas users, women using wood as fuel had an avg. lower mean arterial pressure and diastolic blood pressure at delivery. The conclusion is that combustion products from the burning of biomass fuels are weren linked with a reduced risk | NA | |
| (Mitter et al. 2016) | | Cohort study | Household combustion of fuel including wood in northeastern Iran | NA | None of the fuel type was associated with mortality for respiratory diseases | | Kerosene/diesel fuel as household combustion source, but not wood, was associated with increased mortality for all diseases, CVDs, and IHDs | NA | |
| (Agarwal et al. 2018) | | Cross sectional study | Household combustion of locally acquired wood in open fire cookstove in Kenya | Median levels of CO and PM_2.5_ were at 13.8 ppm (IQR: 7.6-26.9) and 57.0 μg/m^3^ (IQR: 32.0-83.1) respectively | NA | | This study demonstrates that CO and PM_2.5_ levels are high inside homes using traditional cook stoves in western Kenya, and that higher CO levels are associated with both right and left heart abnormalities | NA | |
| (Misra et al. 2018) | | Cross sectional | Biomass combustion in rural South African Women | Self-reported usual fuel (wood vs. electricity) | No significant association was found between wood fuel use and electricity use for respiratory diseases | | No significant association was found between wood fuel use and electricity use for cardiovascular endpoints | No significant association was found between wood fuel use and inflammatory markers | |
| (Clark et al. 2019) | | Cross-sectional study over before and after the intervention | Wood combustion for cooking, improved cook stove interventionin Sichuan China | PM_2.5_: Summer: 74.1 to 51.5 with intervention, 90.7 to 47.3 without;  Winter: 153 to 107 with intervention, 203 to 205 μg/m^3^ without | NA | | Women who did not receive the energy package had greater mean decreases in brachial systolic, DBP compared who received the package. Similar results for central BP, central pulse pressure, and arterial stiffness | NA | |
| (Ruiz-Vera et al. 2019) | | Cross-sectional study in Mexico | Wood smoke exposure vs  liquified petroleum gas (LPG) | WS vs LPG. Exposure was indexed based on length of exposure to wood smoke. | NA | | Expression of plasma miR-126 and miR-155 are significantly higher in those exposed to wood smoke than LPG. Both miR-126 and miR-155 were associated with cardiovascular pathophysiology | NA | |
| (Young et al. 2019) | | Cross-sectional study | Indoor wood combustion for cooking in rural Honduras | 24-h particulate matter (PM_2.5_), black carbon (BC): 126 and 360 μg/m^3^; Justa stove users’ exposures were 66 and 137 μg/m^3^ | NA | | SBP was 2.5 mmHg higher per unit increase in natural log transformed kitchen PM_2.5_ concentration; results were stronger among women 40 years older. Results suggest reduced household air pollution, even when concentrations exceed air quality guidelines, may help lower cardiovascular disease risk, particularly among older subgroups | NA | |
| (Deng et al. 2020) | | Prospective cohort (Chinese longitudinal healthy longevity survey) | Indoor biomass combustion in China | Questionnaire on biomass fuel types (charcoal, wood, straw) | NA | | Reported use of biomass fuel for cooking (50.2%) was associated with a higher risk of hypertension (SBP, DBP, and mean arterial pressure). The association was greater among the oldest people (>=85 yrs. old) | Compared with persistent clean fuel users, participants who reported switching from clean to biomass fuels for cooking had a noticeably higher risk of hypertension | |
| (Sertogullarindan et al. 2012) | | Retrospective review (cohort) | Biomass smoke (mainly during cooking) vs. tobacco smoke exposure in Turkey | Biomass smoke in women vs. tobacco smoke in men | FEV_1_ was lower among men than that in women. | | Pulmonary hypertension was more frequent in women than that in men. | NA | |
| (Golpe et al. 2018) | | Cross sectional | Tobacco smoke vs biomass (mainly for cooking and heating) smoke in Spain | NA | NA | | There was no difference in cardiovascular co-morbidities between biomass exposure and tobacco exposure | NA | |
| **Ambient biomass smoke air pollution** | | | | | | | | | |
| (Sanhueza et al. 2009) | | Ecological time series | Domestic wood combustion caused air pollution in Temuco, Chile | PM_10_: daily avg. range 7-321 with a mean of 46 µg/m^3^ | Increases in PM_10_ were associated with increased hospital admissions for acute respiratory illness | | Increases in PM_10_ were also associated with increased hospital admissions for cardiovascular hospital admissions | The risk was the highest among the elderly | |
| (Arbex et al. 2010) | | Ecological time-series study | Combustion of sugar cane April 23^rd^ to Nov. 30^th^ in 2003 and 2004 in Brazil | TSP (total suspended particles) range from 6.7 to 137.8 μg/m^3^ | NA | A 10 mg/m^3^ increase in the TSP of 3-day moving average and lagged at 1 day led to an increase in hypertension-related hospital admissions during the harvest period that was almost 30% higher than during non-harvest periods | | | NA |
| (Johnston et al. 2013) | Population-level interventional study (ecological) | | Domestic wood combustion for heating. Intervention: education, wood stove replacement in Central Launceston, Australia | PM_10_: annual mean from 23.7 and 18.4, wintertime avg. from 43.6 to 27 µg/m^3^ before and after the intervention | In males the observed reductions in annual mortality were larger and significant for all cause and respiratory diseases | In males, cardiovascular disease mortality reduction was associated with the intervention. Winter-time reductions of PM_10_ was associated with cardiovascular mortality reduction | | | NA |
| (Diaz-Robles et al. 2015) | | Ecological time series | Domestic wood combustion in Chile | The comparison was conducted between cities where one city is using wood combustion and the other was though mobile or point sources | NA | | Increases in PM_10_ were also associated with increased hospital admissions for cardiovascular hospital admissions | Same study as the one Sanhueza et al. 2009 presented | |
| (Yap and Garcia 2015) | | Time series | Residential wood burning | There were reductions of 12%, 11%, and 15% in PM_2.5_, and 8%, 7%, and 11% in coarse particles after implementation of the public health intervention to reduce wood smoke exposure | NA | | Among those aged 65 years and older, Rule 4901 was estimated to prevent 7%, 8%, and 5% of CVD cases, and 16%, 17%, and 13% of IHD cases, in the entire SJVAB and in rural and urban regions, respectively | NA | |
| (Croft et al. 2017) | | Cross sectional study | Wood smoke sourced by delta-C in New York (NY), USA | Delta C levels were positively correlated with PM_2.5_, black carbon, UFP | NA | | An IQR increase in delta C, PM_2.5_, BC, UFP was associated with increased CRP, MPO, and fibrinogen levels, but not with PF4, d-dimer, vWF, or p-selection | NA | |
| (Evans et al. 2017) | | Case-crossover design | Wood combustion as indoor heating source in NY, USA | Delta-c (wood smoke) and carbon black (traffic) was used to estimate the pollutant levels | NA | | ST-elevation myocardial infarction was associated with increases in PM_2.5_, O_3_ in the previous hour, and each 48-h increase in CO. But the association was not significant with only delta-C or black carbon | NA | |
| (Huynh et al. 2018) | | Retrospective cohort study | Wood burning for winter-heating in Tasmania, Australia | Tasmania has excellent air quality with median PM_2.5_ at 2.9 μg/m^3^ | NA | | Increase of PM_2.5_ levels were associated with increase of Heart failure incidence. The association between PM_2.5_ and heart failure hospitalizations was only significant among people who did not take beta-blockers | NA | |
| (Rich et al. 2018) | | Ecological (case-crossover) | Land use in the form of Delta-C in NY, USA | Black carbon: control median 0.69, avg. 1.10 ± 1.25 μg/m^3^; case period median 0.68, and avg. 1.10 ± 1.29 μg/m^3^.  Delta-C: control median 0.1, avg. 0.34 ± 0.64 μg/m^3^; case period median 0.11, and avg. 0.31± 0.57 μg/m^3^ | NA | | ST-elevation myocardial infarction was not associated with changes in black carbon or delta-C concentrations at any time that the study included in the analysis | NA | |
| (Assibey-Mensah et al. 2019) | | Ecological (case-crossover) | Wood combustion in the form of delta-C in NY, USA | PM_2.5_ was weakly correlated with BC (r=0.31) and Delta-C (r=0.26) | NA | | Each 0.52 µg/m^3^ increase in Delta-C concentration during the 7th gestational month was associated with increased odds of hypertensive disorder of pregnancy (HDP) | NA | |
| (Assibey-Mensah et al. 2020) | | Ecological study | Indoor wood combustion for heating in NY, USA | PM_2.5_ concentration and blood delta-c concentration | NA | | Each 3.64 μg/m^3^ increase in fine PM was associated with increased odds of early-onset preeclampsia during the first (odds ratio, 1.35 [95% CI, 1.08–1.68]), second (odds ratio, 1.51 [95% CI, 1.23–1.86]), and third (odds ratio, 1.25 [95% CI, 1.06–1.46]) gestational months. Increases in traffic pollution and woodsmoke during the first gestational month were also associated with increased odds of early-onset preeclampsia | NA | |

**Supplemental Table 3**. Summary of intervention and controlled human exposure studies of wood smoke exposure and cardiovascular effects.

| **Author & year** | **Study type** | **Fuel type** | **Exposure level** | **Pulmonary effects** | | **Cardiovascular effects** | | **Other** | |
| --- | --- | --- | --- | --- | --- | --- | --- | --- | --- |
| (Barregard et al. 2006) | Experimental exposure  (13 subjects) crossover study | 1:1 hardwood and softwood | Mean concentration PM_2.5_ and PM_1_ were 279 and 243 µg/m^3^. Exposure sequence: 4-hour air first and 1 week later 4-h wood smoke | NA | | Factor VIIIc/vWf ratio was increased after wood smoke exposure | | Serum amyloid A (SAA) was significantly elevated after wood smoke exposure. Urine 8-iso-PGF_2a_ excretion was increased after wood smoke exposure | |
| (Allen et al. 2011) | Randomized crossover intervention study | Indoor and outdoor PM_2.5_ and levoglucosan levels were measured for wood smoke exposure in British Columbia, Canada | Measured 7-day levels with or without HEPA filter. Outdoor PM_2.5_ was about 11.2 µg/m^3^. The indoor levels reduced 60% (from 11.2 to 4.6) using HEPA filter | NA | | Endothelial function was measured using peripheral artery tonometry and presented as reactive hyperemia index (RHI). HEPA filters are associated with 9.4% increase of RHI | | Decreased PM_2.5_ levels by HEPA filters are associated with 32.6% decrease in C-reactive protein and band cell counts | |
| (Forchhammer et al. 2012) | Randomized, double-blind, case crossover. 20 subjects (10 male 10 female; 19-55 years old) | Beech wood combusted in a stove | Wood smoke PM_2.5_ at 14, 220, or 354 μg/m^3^. 3-h exposure with 2-week interval. Significantly higher levels of PAHs in the wood smoke. | NA | | Microvascular function (MVF) was not significantly changed after exposure to any of the wood smoke doses | | Wood smoke did not change oxidative stress (HMOX1), inflammation markers (IL-6, CCL2, TNF, IL-8), and DNA damage (OGG1) | |
| (Ghio et al. 2012) | Randomized, double-blind, case crossover | Red oak wood smoke | Exposure level: 485 ± 84 µg/m^3^. 2-h exposure (15 min exercise and 15 min rest) with at least 3-week interval | Lung function was not affected by exposure to wood smoke. There were no respiratory symptoms associated with wood smoke exposure | | 16.8% decrease of maximal heart rate after particle exposure. Coagulation and thrombosis markers, such as vWF, d-dimer, plasminogen, etc were not significantly changed. Autonomic nervous system (HRV)was not affected by the exposure | | Increased neutrophil in lung lavage. Blood IL-1β level was significantly increased following wood smoke exposure | |
| (Stockfelt et al. 2012) | Randomized, case crossover study design | A mixture of hardwood and soft wood (50% birch, 50% spruce) | Wood smoke PM_2.5_ at start-up and burn-out phases were 295 and 146 µg/m^3^, respectively. 3-h exposure without exercise | Fraction of exhaled nitric oxide (FENO), an inflammation marker, increased after wood smoke exposure from the burn-out phase. Exposure also lead to complaints of eye and nose irritation | | NA | | After exposure to wood smoke from the start-up, but not the burn-out session, Clara cell protein 16 (CC16) increased in serum after 4 hours, and in urine the next morning | |
| (Stockfelt et al. 2013) | Randomized, case crossover study design | A mixture of hardwood and soft wood (50% birch, 50% spruce) | Wood smoke PM_2.5_ at start-up and burn-out phases were 295 and 146 µg/m^3^, respectively. 3-h exposure without exercise | NA | | No significant changes were found between wood smoke exposure and systemic inflammation (IL-6, TNFa,) and soluable adhesion molecules (ASS, sP-selection). Only sporadic changes can be found in serum CRP and ICAM1 levels | | NA | |
| (Unosson et al. 2013) | Randomized, double-blind crossover study (14 subjects total) | Birch wood (incomplete combustion) | Target PM_1_ concentration 300 µg/m^3^ for 3 hours. Actual concentration 314±38 µg/m^3^ with intermittent exercise. | NA | | Central arterial stiffness, measured as augmentation index, augmentation pressure and pulse wave velocity, was higher after wood smoke exposure as compared to filtered air. Heart rate, but not blood pressure, was also higher post wood smoke exposure. Wood smoke exposure also led to decreased HRV | | NA | |
| (Bonlokke et al. 2014) | Randomized, double blind, crossover design (20 subjects). | Lab produced: Danish beech wood | Mean exposure level: 13, 222, and 385 µg/m^3^ for filtered air, low, and high exposure levels respectively | Symptoms in the respiratory system was evident | | No significant change of HRV or other related endpoints | | Plasma tissue factor and IL-6 are higher in filtered air group than those in exposure groups. P-selectin declined independent of exposure | |
| (Hunter et al. 2014) | randomized (16 male firefighters) crossover study | Nordic wood stove using birch wood | PM_1_ was about 1000 µg/m^3^. Exposure was about 1 hour with intermittent exercise | NA | | Blood carboxyhemoglobin, forearm blood flow, and vasodilatation to bradykinin were increased following wood smoke exposure compared with clean air. Wood smoke exposure had no effects on arterial pressure, pulse wave velocity, etc | | NA | |
| (Kajbafzadeh et al. 2015) | Interventional study | Unspecified wildfire wood smoke | 6-7 µg/m^3^ without HEPA and 4-5 with HEPA filter | | NA | | No significant relationship was found between PM_2.5_ exposure and endothelial function | | Low level indoor traffic related PM_2.5_ exposure was related to inflammation (increased c-reactive protein) |
| (Muala et al. 2015) same study as Unosson 2013 | Randomized, double-blind crossover study (14 subjects total) | Birch wood, incomplete combustion | Target PM_1_ concentration 300 µg/m^3^ for 3 hours. Actual concentration 314±38 µg/m^3^ with intermittent exercise | NA | | Significant increases in submucosal and epithelial CD3+ cells, epithelial CD8+ cells and submucosal mast cells, were observed after wood smoke exposure | | Significant reductions in macrophage, neutrophil and lymphocyte numbers in BALF were observed following wood smoke exposure | |
| (Andersen et al. 2017) | Human exposure study | Wood smoke with ot without burning mattress. | Total PM: 32 mg/m^3^ in the room. PM number at the breathing zone was 50,000 – 250,000 when not wearing PPE and less than 1,000 particles / cm^3^ when wearing | NA | | Firefighting activity was associated with altered HRV, decreased microvascular function, and increase urine 1-OHP. No difference between wood and wood + mattress | | Personal PPE that firefighters use. Removing PPE during the training was significantly | |
| (Andersen et al. 2018) | Same as above | Same as above | PAH levels on skin was measured as well as 1-hydroxypyrene (1-OHP) to show the metabolite level of PAH in the system. PPE can effectively remove PM from inhalation exposure, but PM exposure happened when the environment was perceived as safe when removing PPE | The subjects who had fire extinction activities during their shift showed decreased lung function | | The subjects who had fire extinction activities during their shift showed increased plasma levels of VCAM1 and reduced levels of oxidatively damaged DNA | | NA | |
| (Fedak et al. 2019) | Controlled human exposure (48 subjects. Each underwent 6 sessions of exposure) | Liquified petroleum gas (LPG) (propane), wood chip burning with different levels of stoves | PM_2.5_ for: LPG 10, Gasifier 35, Fan rocket 100, rocket elbow 250, three stone fire 500 µg/m^3^. 2-hour exposure. | NA | | Thirty minutes after exposure, SBP was lower at the dose of 35 and 500 µg/m^3^ PM_2.5_ compared with the control. Mean SBP was 2 to 3 mm Hg higher for all treatments compared with control except for the dose of 250 µg/m^3^ after 24 hours. No significant changes were found for DBP | | NA | |
| (Walker, Fedak, et al. 2020) | Same as above | Same as above | Same as above | NA | | Pulse wave velocity (PWV), central augmentation index (AIx) were marginally higher 24 hours after all cookstove treatment compared to control | | NA | |

**Supplemental Table 4**. Summary of *in vivo* animal studies on wood smoke exposure and cardiovascular effects.

| **Author & year** | **Study type & strain** | **Fuel type** | **Exposure level** | **Pulmonary effects** | **Cardiovascular effects** | **Other biological markers** |
| --- | --- | --- | --- | --- | --- | --- |
| (Reed et al. 2006) | F344 rats, SHR rats, A/J mice, and C57BL/6 mice | Burning a mixed oak species to generate smoke | Doses: filtered air, 30, 100, 300, and 1000 µg/m^3^. Whole-body inhalation for 6h per day, 7 days a week for either I week or 6 months. Contains both gases and PM | Lung histology showed no sign of wood smoke PM. Parallel studies showed mild effects on BALF parameters and in a mouse model of asthma | No significant changes in any measured parameters related to cardiovascular effects were observed under any exposure condition | Exposure-related effects included increases in platelets and decreases in blood urea nitrogen and serum alanine aminotransferase |
| (Seilkop et al. 2012) | ApoE^-/-^ mouse model (10-week, C57BL/6 background) | Oak | Whole-body inhalation for 6-h per day, 7 days a week for 50 days. Exposure include diesel exhaust and wood smoke | NA. SO_2_, ammonia, NO_x_, and CO were most highly predictive of responses. PM’s role only ranked 3^rd^ to 7^th^ | Endothelial function markers, enthothelin-1, vascular endothelin growth factor, matrix metalloproteinases were not affected by wood smoke exposure | Inflammatory markers as well as oxidative stress marker, HMOX1, were not significantly changed by wood smoke exposure |
| (Kim et al. 2014) | Female CD-1 mice | Peat smoke from smoldering or nearly extinguishing | 100 µg/mouse through oropharyngeal aspiration | No significant lung or systemic effects | Mice exposed to the fire smoke developed significantly decreased cardiac function and greater post-ischemia-associated myocardial infarction | Cytokines neutrophils, and intracellular ROS production in lavage |
| (Aragon et al. 2016) | C57BL/6 mice | Six pollutant including hardwood smoke | 6-h exposure period at PM_2.5_ 380 µg/m^3^, samples were collected 24 h later. Serum was used to incubate primary endothelial cells or aortic rings | NA | Serum from wood smoke exposed mice significantly induced mRNA expression of Vcam1, Icam1. Wood smoke is also the most potent among all to impair vasorelaxation to acetylcholine | Serum from wood smoke exposed mice significantly induced mRNA expression of Il6, Cxcl1 |
| (Martin et al. 2018) | Male Wistar Kyoto rats | Peat smoke generated from an Irish bog | 1 h to filtered air (FA), or low (0.36 mg/m^3^ particulate matter) or high concentrations (3.30 mg/m^3^) of peat smoke in a full-body inhalation chamber | NA | Peat smoke exposure seemed to increase the serum HDL and total cholesterol levels. The exposure also increased heart isovolumic relaxation time | Peat smoke decreased glucose, insulin and HOMA-IR levels. Low peat smoke exposure may render the body more susceptible to mild inflammatory responses |
| (Thompson et al. 2018) | Male SD rats (8 per group) | Peat smoke | 35 µg (Lo PM) or 350 µg (Hi PM) of peat smoke PM extracts, or saline via oropharyngeal aspiration (OA) | High dose had lowest relaxation time of ventilatory responses. | High dose of PM had 45% higher end systolic volume and 17% higher pulmonary artery blood flow acceleration/ejection time ratios. Peat smoke affect the regulation of ventricular ejection and filling volumes | Biomarkers and immune cells in BALF were not different among groups |
| (Farina et al. 2019) | Male BALB/cOlaHsd mice | Biomass smoke extracts (spruce) | Total PAH for wood smoke (50 ± 10 ng/mg). Three repeated doses of 50 µg UFPs /instillation every 3 days | Lung PMN% increase in both UFPs | Significant inflammatory responses (COX-2, MPO) were found in cardiovascular system | Wood smoke induced higher levels of LDH in BALF. Higher oxidative stress was found in DEP treated rats. DEP is more potent than wood smoke |

**Supplemental Table 5.** Summary of *in vitro* studies on wood smoke exposure and biological effects related to cardiovascular system.

| **Author & year** | **Study type & cell type** | **Fuel type** | **Exposure level** | **Biological markers** |
| --- | --- | --- | --- | --- |
| (Liu et al. 2005) | Human pulmonary artery endothelial cells (HPAECs) | Lauan wood (dry wood dust) | The wood smoke contains: 24.8 mg/l particulates. Cells were exposed to 40 µg/ml wood smoke | Wood smoke significantly increased the production of reactive oxygen species (ROS) and mRNA expression of Cu/Zn-SOD and HMOX1, depleted intracellular glutathione. However, it did not affect the mRNA changes of catalase and GPX. The exposure also leads to loss of mitochondrial membrane potential, increase in Bax levels and DNA fragmentation. NAC can reversely rescue the oxidative stress state |
| (Bolling et al. 2012) | Co-culture of A549 and THP-1 cells | Mixture of hardwood (50% birch) and softwood (50% fir) | Significant high level of PAH. Cells were exposed to 40 µg/ml particles either at the start-up (PM_>2.5_) or burn out phase (PM_1.0-2.5_) for either 12 h or 40 hours | Most particle sample exposure significantly increased the release of IL-6, IL-8, and TNF-α from the co-culture at both timepoints. Wood smoke particles, either coarse or fine, significantly induce higher levels of LDH than control, but the level did not max out, indicating relatively low cytotoxicity. The organic fraction was the most important determinant for the WSP-induced effects. But measured PAHs does not necessarily link to the major contributor of the toxicity, indicating other organic chemicals involved. THP-1 cells entered S/G2 phase of the cell cycle, indicating decreased proliferation |
| (Ghio et al. 2012) | BEAS-2B cells | Red oak | Wood smoke particle (100 µg/ml) | Wood smoke particles from smoldering red oak induced significantly increased reactive oxygen species (ROS) using DCFDA assay. Wood smoke did not induce acute cytotoxicity with 24 hours as LDH levels compared to those in control. Wood smoke exposure at either 50 or 100 µg/ml significantly induced increased production of IL-8 |
| (Miousse et al. 2015) | Murine macrophage cell line RAW264.7 | Commercially available wood (pine and oak) | Biomass smoke particulate (5, 50 µg/ml) | Metal component and organic compounds.  Wood smoke induce significant increase in LDH, low-level of cytotoxicity. WS also induce increased mRNA expression of catalase and HMOX1 at 24-h or 72-h compared cells in control. Epigenetic alterations induced by WS treatment was mild |
| (Muala et al. 2015) | Murine macrophage cell line RAW264.7 | Birch wood | 15, 50, 150, 300 µg/ml of the wood smoke extracts | The in vitro data demonstrated that wood smoke particles generated under these incomplete combustion conditions induced cell death and decrease the metabolic activity at high doses (150 and 300 µg/ml). Significant increased number of cells also enter subG1 phase with a concentration-dependent trend. Similar results were found for the DNA damage. But wood smoke exposure only induced minor inflammatory responses (small increase in MIP-2 expression but not TNFα) |
| (Longhin et al. 2016) | Human bronchial epithelial cells (HBC) | Comparative study on DE and biomass smoke (spruce) | Metal and PAH content was higher in diesel samples compared to biomass fuel (2.5 mg/cm^2^) | In this study, only diesel exhaust particles generated more mRNA expression responses in xenobiotic metabolism, inflammatory response, oxidative stress, and EMT after 24 h or 2-week exposure at the same mass dose. Similarly, more pro-inflammatory proteins (IL6, IL-8, IL-1β) were released in the cells treated with DE compared to wood smoke at 24h, 1-week, or 2-week |
| (Arif et al. 2017) | A549 and BEAS-2B | Miscanthus straw, softwood chips (SWC spruce), hard wood chips (HWC beech) | PAH and CO: Beech > softwood > miscanthus. TVOCs: softwood generate highest levels.  Exposure doses: 0, 1, 3, 10, 30, 100 µg/cm^2^. | SWC and BWC can cause significant cytotoxicity at higher doses at 30 and 100 µg/cm^2^ in both cell lines. SWC and BWC can cause significant cellular ds-DNA damages at higher doses at 10, 30 and 100 µg/cm^2^ in both cell lines. SWC and BWC can cause significant necrosis and apoptosis at higher doses at 30 and 100 µg/cm^2^ in BEAS-2B cells. Particle bound PAHs in WCPM_0.4-1_ and BWCPM_0.4-1_ are most likely the cause for the toxic effects seen in the human lung cells studied. Calcium and selenium are found positive correlated to the toxicological endpoints in both cell lines |
| (Grilli et al. 2018) | BEAS-2B | Spruce. Compared with diesel exhaust | 20 h exposure to UFP at 25, 50, and 100 µg/ml. Time course was at 25 µg/ml for 1, 4, 8, 16, and 30 hours. | The exposure to ultrafine particles from biomass determines less distinct modifications of the gene expression profiles. Less strong association compared to diesel UFPs, biomass UFP exposure induces the secretion of biomarkers associated to inflammation (CCXL2, EPGN, GREM1, IL1A, IL1B, IL6, IL24, EREG, VEGF) and transcription factors (as NFE2L2, MAFF, HES1, FOSL1, TGIF1) relevant for cardiovascular and lung disease |
| (Zeglinski et al. 2019) | A549 | Birch wood | 5% or 10% wood smoke | No acute cytotoxicity of the wood smoke. WS exposure resulted in a significant reduction in barrier function with significantly decreased junction protein (E-cadherin) levels dependent with increasing doses. WS exposure induced activation of the p44/42, but not p38, MAPK signaling pathway, and inhibition of p44/42 phosphorylation |
